# Quantum Chemical Tailoring of Donor–Acceptor Organic Nanomedicines for Nonlinear Optical Performance and Theragnostic Applications: Molecular Descriptors, In Silico, and Ab Initio Investigations

**DOI:** 10.3390/ijms27167468

**Published:** 2026-08-20

**Authors:** Sehar Nadeem, Muhammad Usman Khan, Łukasz Szeleszczuk, Dariusz Maciej Pisklak, Marcin Gackowski, Salah Knani, Nadia Ayari

**Affiliations:** 1 Department of Chemistry, University of Okara, Okara 56300, Pakistan; sehar.nadeem230@gmail.com; 2 Department of Organic and Physical Chemistry, Medical University of Warsaw, 1 Banacha Str., 02-097 Warsaw, Poland; dpisklak@wum.edu.pl; 3 Department of Toxicology and Bromatology, L. Rydygier Collegium Medicum in Bydgoszcz, Nicolaus Copernicus University in Torun, 2 Jurasza Str., 85-089 Bydgoszcz, Poland; marcin.gackowski@cm.umk.pl; 4 Center for Scientific Research and Entrepreneurship, Northern Border University, Arar 73213, Saudi Arabia; saleh.kenani@nbu.edu.sa; 5 Department of Physics, College of Science, Northern Border University, Arar 91431, Saudi Arabia; nadia.alayari@nbu.edu.sa

**Keywords:** photothermal therapy, D–π–A chromophores, NLO, nanomedicine

## Abstract

Nonlinear optical (NLO) active chromophores and their applications in photothermal therapy (PTT) demonstrate great potential in modern theragnostics, owing to their efficient light–matter interactions. A recognized D–π–A chromophore, FTC-3f, which is reported to exhibit a high photothermal conversion efficiency (~51.11%), was structurally modified with various spacers and acceptors to elucidate enhanced PTT and NLO behavior through DFT and TD-DFT simulations. Molecular geometries were optimized at the B3LYP/6-31G (d, p) level. Their electronic properties, charge separation, and efficient transition pathways were analyzed using frontier molecular orbitals (FMOs), density of states (DOS), UV–visible spectroscopy, photon-induced electron transfer (PET), and transition density matrix (TDM) analysis. Among all the derivatives, D4 exhibited the smallest HOMO–LUMO energy gap (1.701 eV). The highest first-order hyperpolarizability (β) values are found for D4 (1.50 × 10^5^ in the gas phase, 7.40 × 10^5^ in water, 3.06 × 10^5^ in benzene solvent). The β_HRS_ is found to be in the range 5.11 × 10^4^ to 2.97 × 10^4^ and DR (3.65 × 10^5^ to 4.53 × 10^4^) supports the strong NLO response and strong synergistic donor–acceptor interactions. The designed chromophores exhibit much higher SHG and EOPE responses at 532, 1907.21, and 1064 nm than FTC-3F, indicating great potential for the synthesis of effective NLO nanomedicine. Molecular docking with bovine serum albumin (PDB ID: 4F5S) and Bcl-2 (PDB ID: 2W3L) suggested favorable binding conformations, consistent with a potential for transport and apoptotic-targeting behavior. All 3D molecular descriptor parameters indicate that the designed chromophores have potential for preferential targeting in PTT. This study investigates how structural modifications of donor–π–acceptor chromophores influence their electronic, optical, nonlinear optical, and theragnostic properties, providing insights into molecular design strategies for advanced NLO-based nanomedicine applications.

## 1. Introduction

Near-infrared (NIR) photothermal agents can absorb NIR irradiation and convert it into thermal energy by avoiding radiative relaxation in materials, demonstrating significant potential for photothermal therapy (PTT) [1,2,3,4,5]. A large number of NIR photothermal agents have been developed, including metal sulfides [6,7], organic compounds [8,9], carbon nanomaterials [10,11], and gold nanostructures [12,13], to name a few. Due to their excellent biocompatibility, high chemical flexibility, and ease of assessing structural–property relationships, organic π-conjugated compounds have attracted considerable interest among photothermal agents [14,15,16]. Various commonly used chemical reagents, such as indocyanine green [17] and methylene blue [18], are FDA-approved for use in clinical settings. In general, however, conventional organic compounds exhibit pronounced photobleaching or inefficient non-radiative decomposition under light, as expected [19,20]. Thus, they cannot be the most suitable agents of PTT. The new ideas of creating new molecular designs of advanced organic compounds with consistent photothermal potential for PTT and efficient non-radiative decay are quite desirable. Nonlinear optical (NLO) chromophores are electron donors, π-conjugated bridges, and strong electron acceptors (D–π–A) and have increasingly become a large family of organic compounds [21,22,23,24]. It is worth noting that these NLO chromophores could exhibit high chemical stability and photothermal efficiency through appropriate structural tailoring, offering great potential for use in PTT [19,25,26]. Recently, to produce PTT, a D–π–A structure containing an NLO chromophore has been reported. As shown by the experimental data, the synthesized chromophore exhibits very high stability under physiological and high-temperature conditions, as well as a high photothermal conversion efficiency of approximately 52.71% [27]. Yet, effective chromophore-based photothermal therapy (PTT) is achieved under 635 nm laser irradiation, which severely limits its effectiveness due to the shallow penetration of light in tissues. The fabrication of nanostructures using NLO chromophores that absorb near-infrared (NIR) light is highly attractive for photothermal therapy (PTT), as NIR offers greater penetration, sensitivity, and resolution than visible light. This was followed by the synthesis of a near-infrared-absorbing organic nanoparticle by the self-assembly of thermally stable nonlinear optical chromophores using the amphiphilic polymer methoxy poly(ethylene glycol)-poly(lactide-co-glycolide) (mPEG-PLGA). It has an NLO chromophore with a D–π–A structure that exhibits broad near-infrared absorption and strong absorption. When the NLO/PLGA nanoparticles were encapsulated in mPEG-PLGA, they not only retained a high level of absorption in the NIR region but also showed a great deal of stability when suspended in water, phosphate-buffered saline (PBS), and Dulbecco’s modified Eagle’s medium (DMEM). NLO/PLGA exhibited a photothermal conversion efficiency of 51.11%, good photoacoustic behavior, and remarkable photothermal stability in tumors under NIR irradiation, reflecting great potential for noninvasive photoacoustic imaging and noninvasive photothermal therapy of tumors. This paper describes the nanoparticle 2(3-cyano-4(E)-2(5-((E)-4(dibutylamino)styryl)thiophen-2-yl)vinyl)-5(5-phenylthiophen-2-yl)-5(trifluoromethyl)-furan-2(5H)-ylidene)-malononitrile, with a D–π–A standard configuration being determined, where different NLO chromophores are used with varying π-spacers and acceptors. The reference (FTC-3F) chromophores comprise the N, N-dibutyl-4- vinylaniline donor group, thiophene as a π-spacer, and a 2(3-cyano-5(5-phenylthiophen-2-yl)-5(trifluoromethyl)-4-vinylfuran-2(5H)-ylidene) malononitrile acceptor group [28]. We have designed the D molecule using the same donor and acceptor components, along with a thiazole spacer. Four new molecules are then prepared using the acceptor moieties D1 and D2, designed by adding -CF3 and -NO2 fragments to FTC-3F, and D3 and D4, which also incorporate -CF_3_ and -NO_2_ fragments into the D molecule. Spacer and acceptor fragments were intended to improve linear and nonlinear optical properties.

The main aim of this research is to investigate the structure–property relationships governing the electronic, optical, nonlinear optical (NLO), and theragnostic properties of various donor–π–acceptor organic chromophores through rational molecular engineering. We hypothesize that systematic variation of the π-conjugated spacer and electron-accepting groups can regulate intramolecular charge transfer (ICT), thereby reducing the HOMO–LUMO energy gap, increasing the extent of light absorption in the visible/near-infrared region, and, therefore, enhancing the nonlinear optical response and potential for biological interaction. To test this hypothesis, some structural modifications have been introduced to the reference FTC-3F molecule and thoroughly explored by density functional theory (DFT) and time-dependent DFT (TD-DFT). The frontier molecular orbitals (FMOs), density of states (DOS), transition density matrix (TDM), localized orbital locator (LOL), natural bond orbital (NBO), and UV–Vis calculations were used in conjunction with molecular descriptors and molecular docking studies to understand the link between molecular structure, charge transfer behavior, electronic properties, and possible theragnostic applications. Furthermore, to investigate the biointeraction potential of the designed FTC-3f derivatives for photodynamic therapy, molecular docking was performed. Bovine serum albumin (BSA, PDB ID: 4F5S) was used to study the translocation and bioavailability of the photosensitizer [29], whereas Bcl-2 protein (PDB ID: 2W3L) was used to study the PDT-induced apoptotic interactions [30]. Additionally, this molecular engineering strategy offers new opportunities to develop effective NLO-active chromophores for optical technologies and photoacoustic (PA) imaging applications.

## 2. Results and Discussion

**Figure 1:**
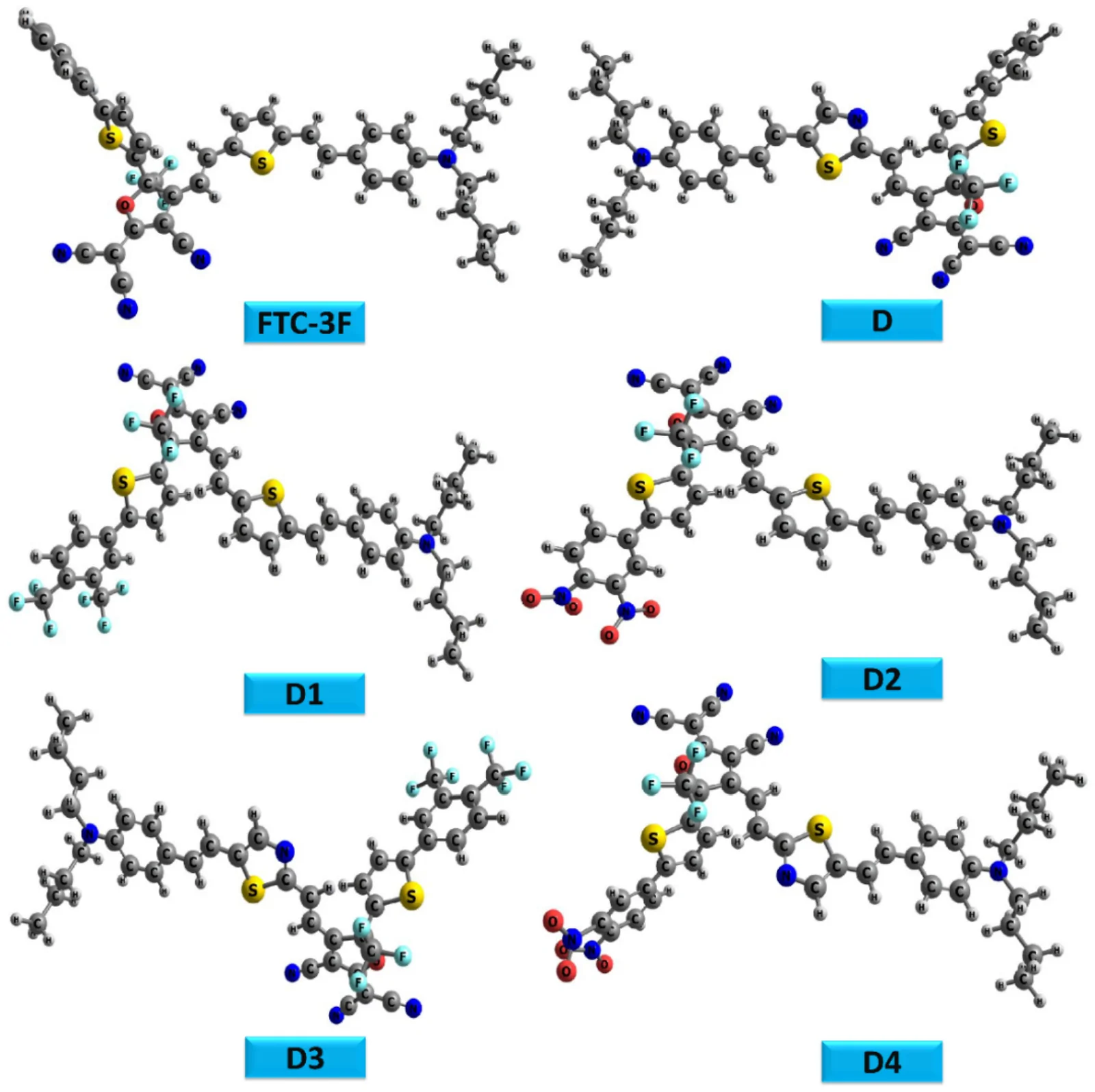
Optimized geometries of the designed NLO-active chromophores.

**Figure 2:**
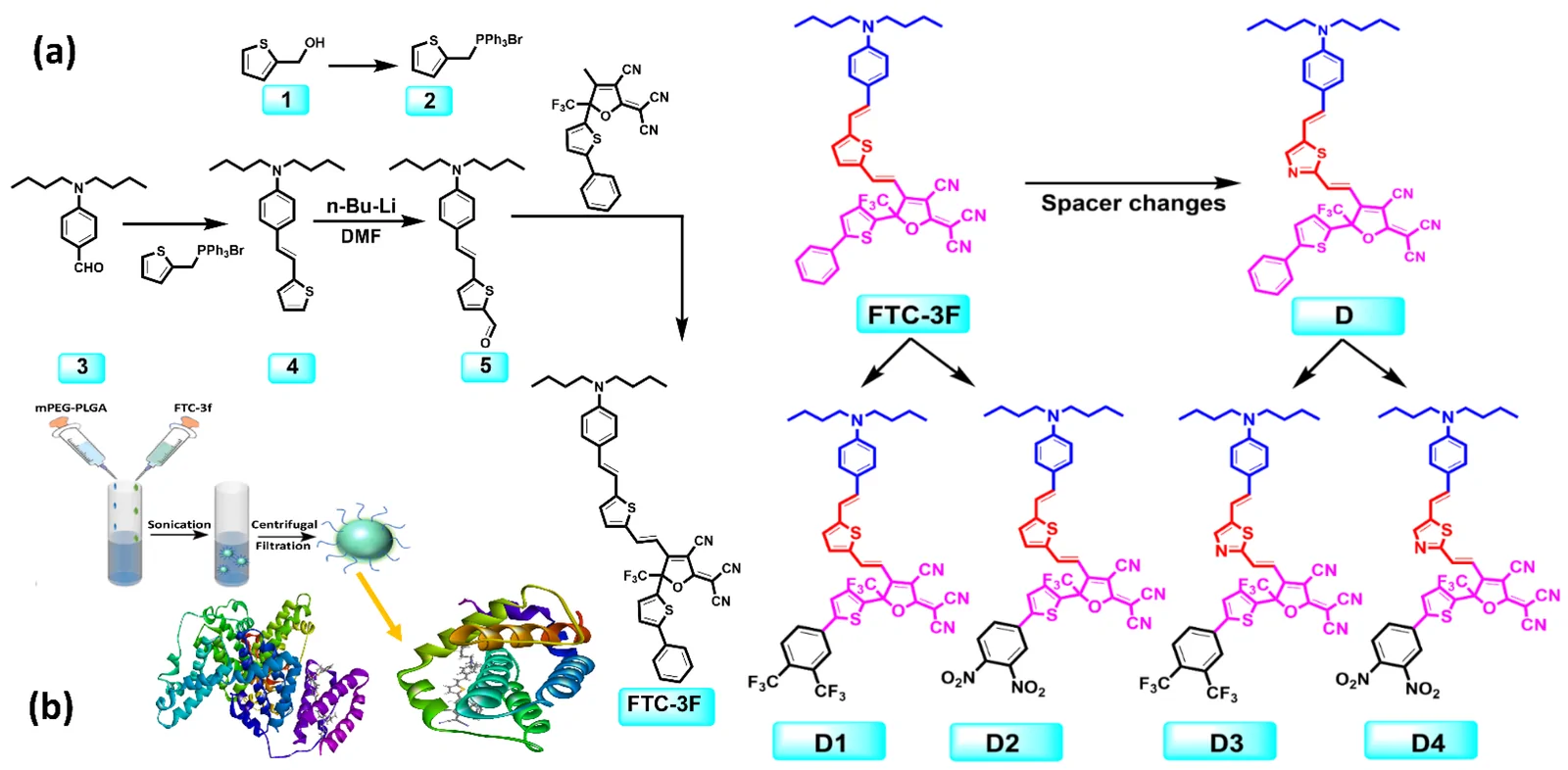
(**a**) The synthetic route for the synthesized reference compound (FTC-3F), and (**b**) the in vitro strategy. The theoretically designed chromophores under investigation (**on the right**).

The feasibility design of the NLO chromophore is an effective approach to acquire low-bandgap organic materials. In prior studies, an experimentally synthesized D–π–A type organic chromophore, named FTC [31], was synthesized with the use of N, N-dibutylaniline as a donor, planar thiophene ring as a π-conjugation bridge, and 2-dicyanomethylene-3-cyano-4-methyl-2,5-dihydrofuran (TCF) as an acceptor. The chromophore FTC has a broad, intense absorption band at 600–700 nm. To shift the absorption band from the visible to the NIR region, a CF3-TCF acceptor with high electron-withdrawing capacity is added to the chromophore [32]. The obtained FTC-3f NLO chromophore exhibited strong absorption in the spectral range of 600 to 1100 nm, with a maximum at 728 nm. Hence, the FTC-3f chromophore can be used in NIR light-activated theranostic biomedicine [28]. Based on this modification, we have screened various electron-withdrawing groups (CF_3_ in D1, NO_2_ in D2) to optimize the donor’s acceptor capacity for charge withdrawal. As we have stated previously, the thiazole-based spacer gave greater improvement towards the NLO characteristics [33]. That is why we have designed the D3 compound, which contains a thiazole and a CF3 acceptor group, and the D4 compound, which contains a thiazole spacer and an NO_2_ fragment. Figure 1 represents the optimized molecules, and their counter 2D illustration is in Figure 2. The electronic, optical, and nonlinear optical properties are analyzed using a DFT and TD-DFT study.

Nonetheless, the hydrophobicity and short circulation half-life of free NLO chromophores remain major challenges to their application as bioavailable agents. To avoid such impediments, scientists coat NLO chromophores with mPEG-PLGA, an FDA-approved polymer with PLGA and PEG as the hydrophobic and hydrophilic terminal groups, respectively, via a nanoprecipitation method. The in vitro research was conducted based on the schematic representation of the encapsulating procedure shown in Figure 2 and as reported in the literature [32]. To determine the biointeraction capability of FTC-3f relative to photodynamic treatment, we performed in silico molecular docking of the developed chromophores. Bovine serum albumin (BSA, PDB ID: 4F5S) was used to study the transport and bioavailability of the photosensitizer, and the Bcl-2 protein (PDB ID: 2W3L) was used to analyze PDT-induced apoptotic interactions. The docking results indicated a favorable predicted binding pose of the compound within hydrophobic cavities, due to the formation of hydrogen bonds and π–π interactions, supporting the usefulness of FTC-3f as a photoresponsive bioactive NLO material.

### 2.1. DFT Study

#### 2.1.1. FMO Analysis

**Figure 3:**
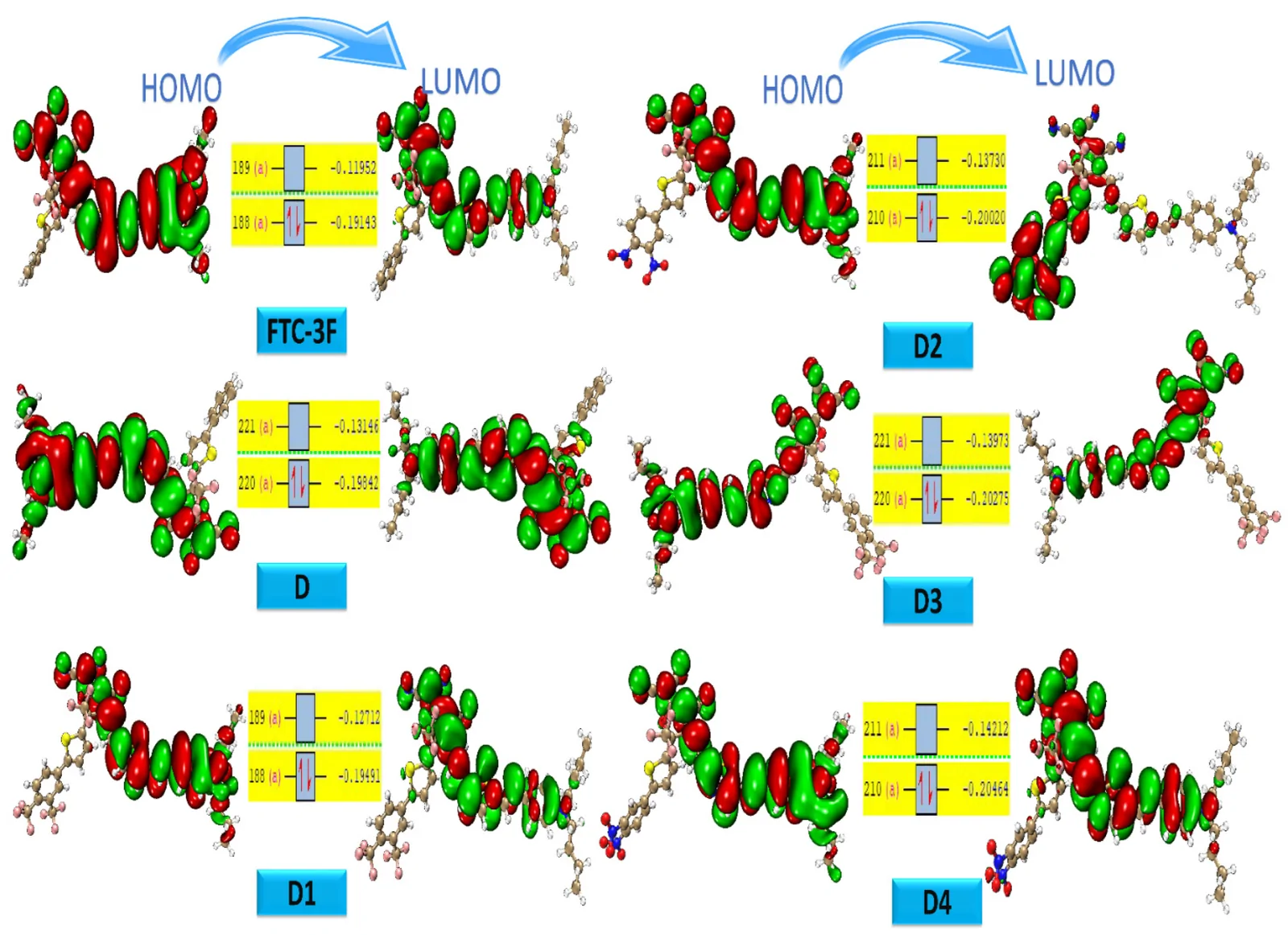
The frontier molecular orbitals on all investigated molecules (D, D1–D4).

The frontier molecular orbitals (FMOs) are computed to understand the optoelectronic, molecular, and electrical structure of chromophores. In particular, the energies and spatial distributions of the lowest unoccupied molecular orbital (LUMO) and highest occupied molecular orbital (HOMO) can be used to investigate intramolecular charge transfer (ICT), optical transitions, polarizability, and nonlinear optical (NLO) responses [34,35]. Greater NLO capability and higher charge mobility are generally associated with a smaller energy gap (Eg), which is favorable for theragnostic and photonic applications. DFT calculations at the B3LYP/6-31G(d,p) level of theory were used to perform FMO analysis of the computationally prepared derivatives D, D1, D2, D3, and D4, as well as the experimentally synthesized chromophore (FTC-3F) in the current study. All molecular geometries were optimized before electronic structure analysis to reach the lowest attainable energy states. Table S1 (Supplementary Materials) summarizes the corresponding orbital energies and energy gaps, and Figure 3 displays three-dimensional iso-density plots of the HOMO and LUMO for each of the three molecules studied. FTC-3F has been shown to exhibit a moderate donor–acceptor (D→A) interaction, with the HOMO largely localized to the donor segment and the LUMO largely evenly distributed across the acceptor moiety. This spatial separation, unlike the planned ones, encourages intramolecular charge transfer upon excitation; however, the relatively large energy separation (Eg = 1.925 eV) indicates that little electronic delocalization is expected. The introduction of a thiazole spacer into the proposed molecule D enhances π-conjugation, thereby improving electronic communication between donor and acceptor units. As a result, a noticeable reduction of the HOMO (1.833 eV) is observed in the HOMO (1.833 eV) to LUMO gap. The electron density of LUMO expands over the acceptor fragment via the thiazole spacer, enabling effective ICT, while the HOMO density spreads over the donor fragment. Other structural modifications to D1 and D2 enhance the D A structure, thereby further stabilizing the LUMO energy levels. Consequently, Eg exhibits better charge transfer properties, with values of 1.822 eV towards D1 and 1.712 eV towards D2. The delocalization pattern in these molecules confirms a substantial difference in electron density between the donor part of the HOMO and the acceptor part of the LUMO, which contributes to higher optical polarizability. The most significant electronic alteration is observed in D3 and D4, which are analogs of D with very strong electron-withdrawing substituents (NO2 and CF3, respectively). The LUMO in such systems is strongly delocalized around the acceptor region and electron-withdrawing groups, and the HOMO is generally highly concentrated on the donor framework. The presence of NO_2_ and CF_3_ groups in the molecule lowers the LUMO energy more effectively, creating small energy gaps of 1.715 eV (D3) and 1.701 eV (D4).

The ICT and electronic polarization are high in D4 due to a strong -I (inductive effect) and -M (mesomeric effect) upon CF3 addition, and it shows the lowest energy gap in the designed series. The overall trend in the HOMO–LUMO energy gap is the following: D4 < D2 < D3 < D1 < D < FTC-3F. The decreasing energy trend shows that the molecular substitution of spacer and acceptor fragments is effective. The enhanced intramolecular charge transfer and reduced band gaps in the proposed derivatives, particularly D3 and D4, considerably support their outstanding nonlinear optical responses. The electrical properties make the proposed chromophores good candidates for organic nanomedicine-based theragnostic applications, in which robust NLO activity and high light–matter interaction are important.

#### 2.1.2. Density of State Analysis

**Figure 4:**
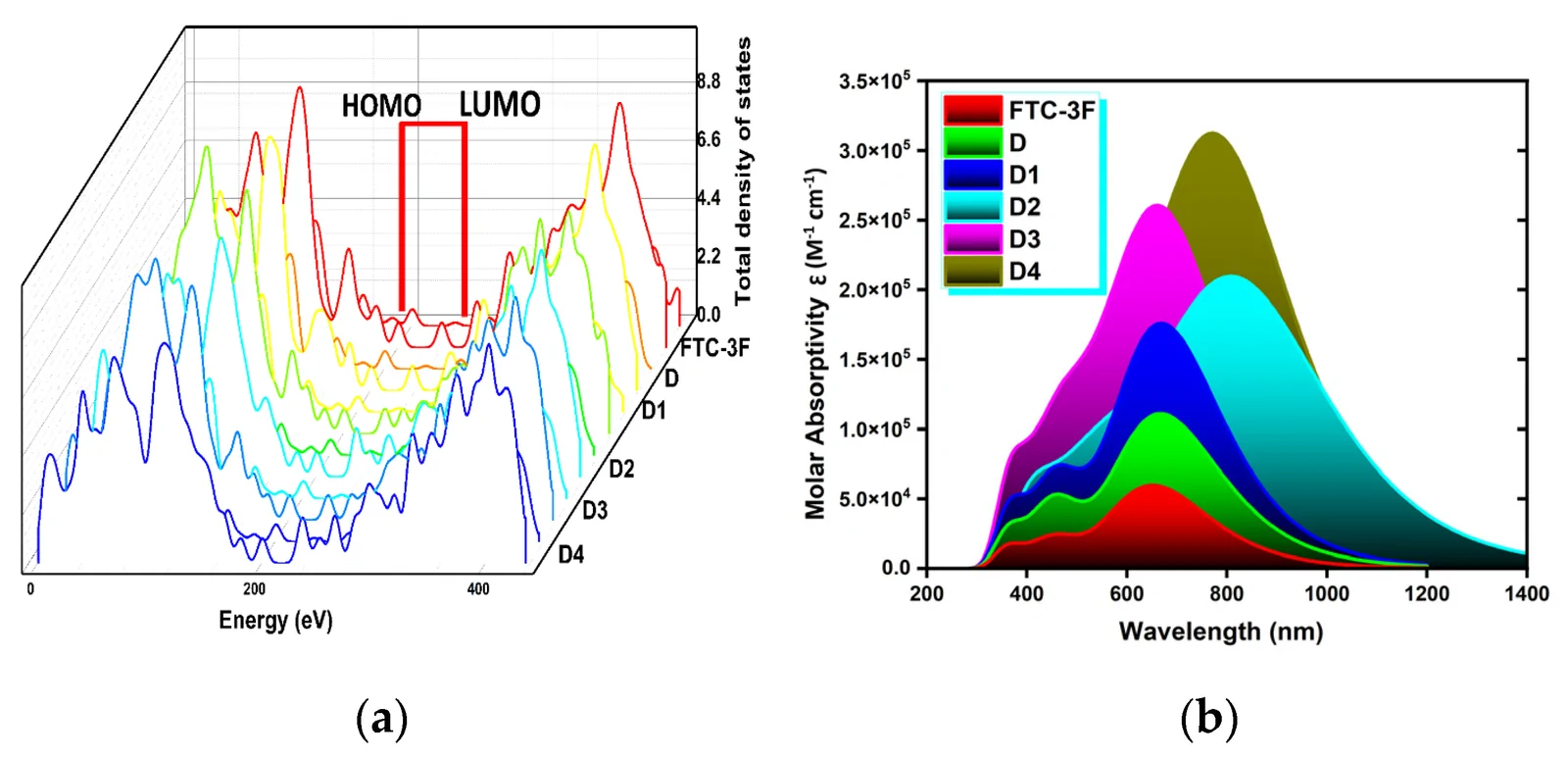
(**a**) The total density of states in all designed compounds due to their donor, acceptor, and spacer fragments. (**b**) The optical absorption in all investigated compounds in the UV–Vis range.

The DOS analysis was conducted to support further the results of the frontier molecular orbital (FMO) analysis and to understand better the roles of the donor, spacer, and acceptor fragments in the electronic structure of the examined chromophores [36]. DOS provides a quantitative account of the electron density distribution around the HOMO and LUMO levels and is important for understanding intramolecular charge transfer (ICT), which is crucial to nonlinear optical (NLO) activity [37]. The percentage composition of different fragments of all designed compounds in HOMO and LUMO is given in Table S2 (Supplementary Materials). In Figure 4a, the DOS plots of FTC-3F and proposed molecules (D, D1–D4) are presented, and the contribution of each fragment is indicated in the table. The HOMO of the reference molecule (FTC-3F) is mainly centered on the donor fragment (67%), the spacer fragment (15.4%), and the acceptor fragment (17.6%). This implies that the density of electrons in the ground state is mainly donor-centered. Conversely, the acceptor unit also has a considerable share of the LUMO (64.4%), which shows that charge transfer between the donor and the acceptor after excitation is successful. The intramolecular charge density is allowed due to charge transfer between the donor and acceptor. The same trend is observed in the proposed molecule D, with the donor contributing 72.28% in the HOMO and the acceptor playing the LUMO (65.5%), with a significant spacer contribution (21.48%). This distribution shows that the thiazole spacer assists the donor and acceptor units in electronic transition. In D1, the acceptor makes a relatively greater contribution to the HOMO (20.5%), indicating greater conjugation, although the donor still dominates the HOMO (63%). The LUMO of D1 remains acceptor-centered (61%), which shows further ICT performance. It is worth noting that D2 has a distinct DOS distribution, in which the spacer (1.1%) and donor (0.8%) contribute negligibly to the LUMO, which is predominantly localized on the acceptor fragment (98.1%). Substantial electron-withdrawing properties are indicated by this high acceptor dominance, which leads to highly effective charge transfer and improved electronic polarization. The contributions to the acceptor moiety (63.4%) and spacer (21.8%) in D3 are again attributed to the LUMO, indicating successful charge delocalization throughout the conjugated molecule. HOMO contribution, on the other hand, is once more dominated by the donor fragment in D3. The high electron-withdrawing power of the CF_3_ group is seen in D4, where LUMO is the highest contributor of the acceptor among the majority of the derivatives (71.5%), with HOMO being largely contributed by the donor (69.1%). This greater acceptor involvement in the LUMO reinforces ICT behavior, thereby strongly promoting donor-to-acceptor electron migration. Overall, the DOS analysis supports the idea that donor fragments dominate the HOMO levels, while acceptor units dominate the LUMO levels across all examined chromophores. The TDOS analysis in Figure 4a shows that the occupied states are primarily concentrated in the HOMO region. In contrast, the unoccupied states are in the LUMO region, separated by a distinct energy gap. The total density of states provides a detailed description of the energetic alignment and electronic states, which is vital in the study of intramolecular charge transfer (ICT) and nonlinear optical (NLO) behavior.

#### 2.1.3. UV–Visible Analysis

UV–vis spectroscopy is the most important spectroscopic technique, elucidating the optoelectronic properties of molecular systems, including electronic transitions and excitation dynamics, and their applicability to nonlinear optical (NLO) studies [38]. The UV–Vis absorption spectra of the nm-FTC-3F reference chromophore and the designed derivatives (D, D1–D4) were calculated at the B3LYP/6-31G (d, p) level of theory in the gas phase in the present study. It is a cost-effective approach that enables measurement of an oscillator’s vibrational strengths, charge transfer characteristics, excitation energy, and maximum absorption. All transition data are summarized in Table S3a (Supporting Information), and the simulated UV–Vis spectra for all systems under study are plotted in Figure 4b. The strong absorption band at 653.34 nm in the visible region of the UV–Vis spectra of FTC-3F is attributed to a HOMO → LUMO electronic transition with a dominant contribution of 100% and an oscillator strength of 1.4617. This favorable red-to-blue shift reveals efficient intramolecular charge transfer (ICT) in the donor–acceptor structure of the reference molecule. A bathochromic shift is observed in the proposed molecule D, with the highest absorption at 688.07 nm. The thiazole spacer introduces improved π-conjugation, which causes this red shift. The transition is mostly controlled by a HOMO → LUMO excitation with a significant oscillator strength of 1.2979. The UV–Vis spectrum of D1 exhibits further red shifting, with a significant absorption peak at 673.90 nm that corresponds to a HOMO → LUMO transition with an oscillator strength of 1.5868. Similarly, D2 has its highest absorption at 677.62 nm, due to a HOMO→LUMO+1 (97%) transition with the highest oscillator strength among all the compounds studied (*f* = 1.6042). The enhanced intensity and extent of the bathochromic shift, and the strong absorption of D1 and D2, are indicative of stronger ICT properties and enhanced interaction between the donor and acceptor. The most evident bathochromic changes are exhibited by the chromophores D3 and D4 that possess the maximum absorption of 713.04 nm and 715.43 nm, respectively. HOMO dominates these transitions to the LUMO, with large oscillator strengths of 1.4248 (D3) and 1.4212 (D4). The fact that strong electron-withdrawing substituents (NO_2_ in D3 and CF_3_ in D4) effectively stabilize the LUMO and enhance charge transfer on excitation makes these strong electron-withdrawing substituents responsible for the large red shift in these molecules. All the designed chromophores exhibit systematic bathochromic shifts and a high absorption band in the visible range compared with the reference nm-FTC-3F. The higher donor–acceptor interaction and high intramolecular charge transfer (ICT) confirm the spectral characteristics required to enhance nonlinear optical performance and photonics. Consequently, the chromophores being studied, in particular D3 and D4, are very promising for NLO-based theragnostic applications.

#### 2.1.4. PET Analysis

**Figure 5:**
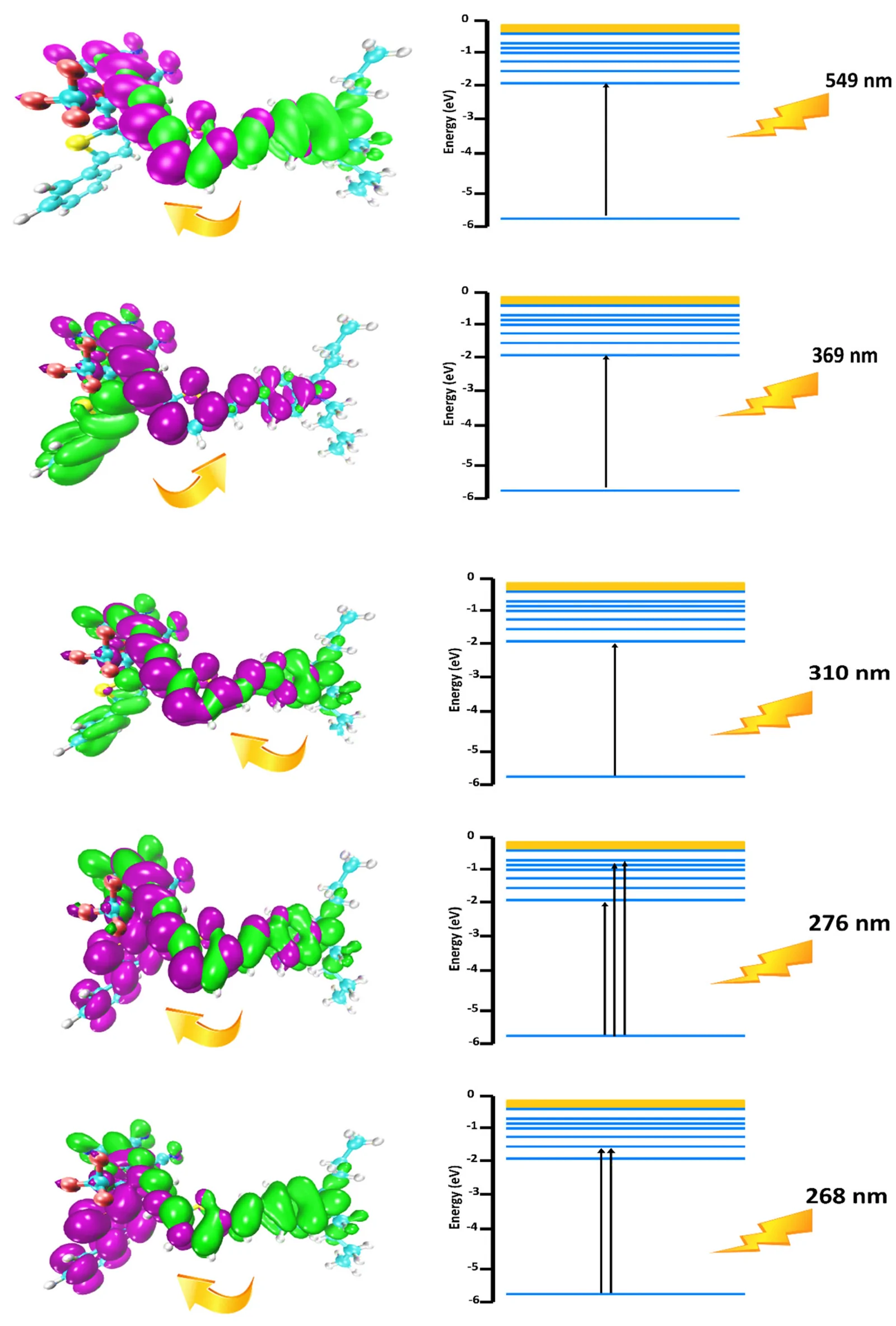
Electron–hole orbital diagram along with the photon-induced diagram of the electron of FTC-3F.

Systematic studies of the photoinduced electron transfer (PET) properties of the reference chromophore FTC-3F (C_41_H_35_F_3_N_4_OS_2_) and its designed molecules D (C_40_H_34_F_3_N_5_OS_2_), D1 (C_43_H_33_F_9_N_4_OS_2_), D2 (C_41_H_33_F_3_N_6_O_5_S_2_), D3 (C_42_H_32_F_9_N_5_OS_2_), and D4 (C_40_H_32_F_3_N_7_O_5_S_2_), distributions of electrons and holes, and excitation diagrams are illustrated in Figure 5. This analysis explains that the bioactivity of the investigated molecules is studied through electron transitions in excited states [39].

In the case of the absorptive processes, electron–hole distribution diagrams have been drawn of the initial five excited states. The details of the transitions and pattern of excitation are shown in Figure 5, and the calculated excitation energy (E_ext_), absorption wavelengths (λ), and oscillator strengths (*f*) are tabulated in Table S3b. The maximum absorption of the reference FTC-3F lies at 549 nm, whereas the designed derivatives are red-shifted with D, D1–D4 values of 560, 584, 590, 604, and 609 nm, respectively [40]. This positive bathochromic shift indicates increased conjugation and improved intramolecular charge transfer resulting from structural changes. In Figure 5, the hole and electron density molecular orbitals are denoted in magenta and green, respectively. In the case of FTC-3F, the electron–hole distribution is rather localized around the conjugated backbone, which implies that there is not much charge separation. But with a structural adjustment, the derivatives show a progressive spatial separation between the electron and hole densities. The thiophene derivatives (D1 and D2) have moderate delocalization along the π-system, and the thiazole derivatives (D3 and D4) have strong charge separation with the density of holes almost on the unit of the donor and the density of the electrons relocated on the unit of the acceptor. This increased separation implies better PET efficiency and a low probability of recombination. To give a more comprehensive analysis, the stabilization energies and the donor–acceptor interactions are provided in Table S4 (Supplementary Materials). The energy E^2^ of the second-order perturbation reflects the degree of intramolecular charge transfer. The reference molecule exhibits intermediate stability, whereas the prepared derivatives exhibit enhanced interactions due to an augmented spacer and stronger acceptor groups. It is important to note that strong lone-pair (O) to π* interactions cause D2 to have a much higher stabilization energy (143.86 kcal mol−1), indicating high charge delocalization. Nonetheless, the stabilization energies of D3 and D4 are equal and are preferable in controlled charge transfer processes. The drop in the excitation energies of FTC-3F from D4 further explains the improved electronic stability and a smaller energy gap. This is due to structural changes such as the addition of nitrogen and fluorine and the addition of thiazole spacers, which improve electron-withdrawing capability and conjugation length. Consequently, the efficiency of PET and its stability in the following sequence are seen: D4 > D3 > D2 > D1 > D > FTC-3F. Generally, the increase in charge separation and effective electron transfer in the designed derivatives, especially D3 and D4, indicates strong PET behavior. This helps in nonradiative decay pathways that produce fluorescence quenching, which is very useful in photothermal therapy (PTT) applications. Also, the enhanced intramolecular charge transfer properties imply that these chromophores have great potential for nonlinear optical (NLO) applications. These results indicate that rational molecular-level structural change is a successful strategy to tune the unbiased PET dynamics that make D3 and D4 effective for future applications in photothermal therapy (PTT) with high absorption efficiency (long-wavelength) and efficient nonradiative decay. Moreover, the increased intramolecular charge transfer in these systems is beneficial for nonlinear optical (NLO) applications, indicating that they can serve as high-performance optoelectronic materials.

#### 2.1.5. Topological Analysis

Topology analysis based on DFT, including TDM and LOL, is used to study intramolecular interactions and the physical characteristics of the designed dyes. The parameters are calculated to investigate the data using the B3LYP/6-31G(d,p) level. Multiwfn 3.8 and the VMD code were used for TDM and LOL analyses; the former is a comprehensive program for analyzing and visualizing wave functions. Topological studies of the localized orbital locator (LOL) and transition density matrix (TDM) are used to investigate covalent bonds and charge distributions and to highlight regions of the molecule that contain a large number of electron pairs.

#### 2.1.6. LOL Analysis

**Figure 6:**
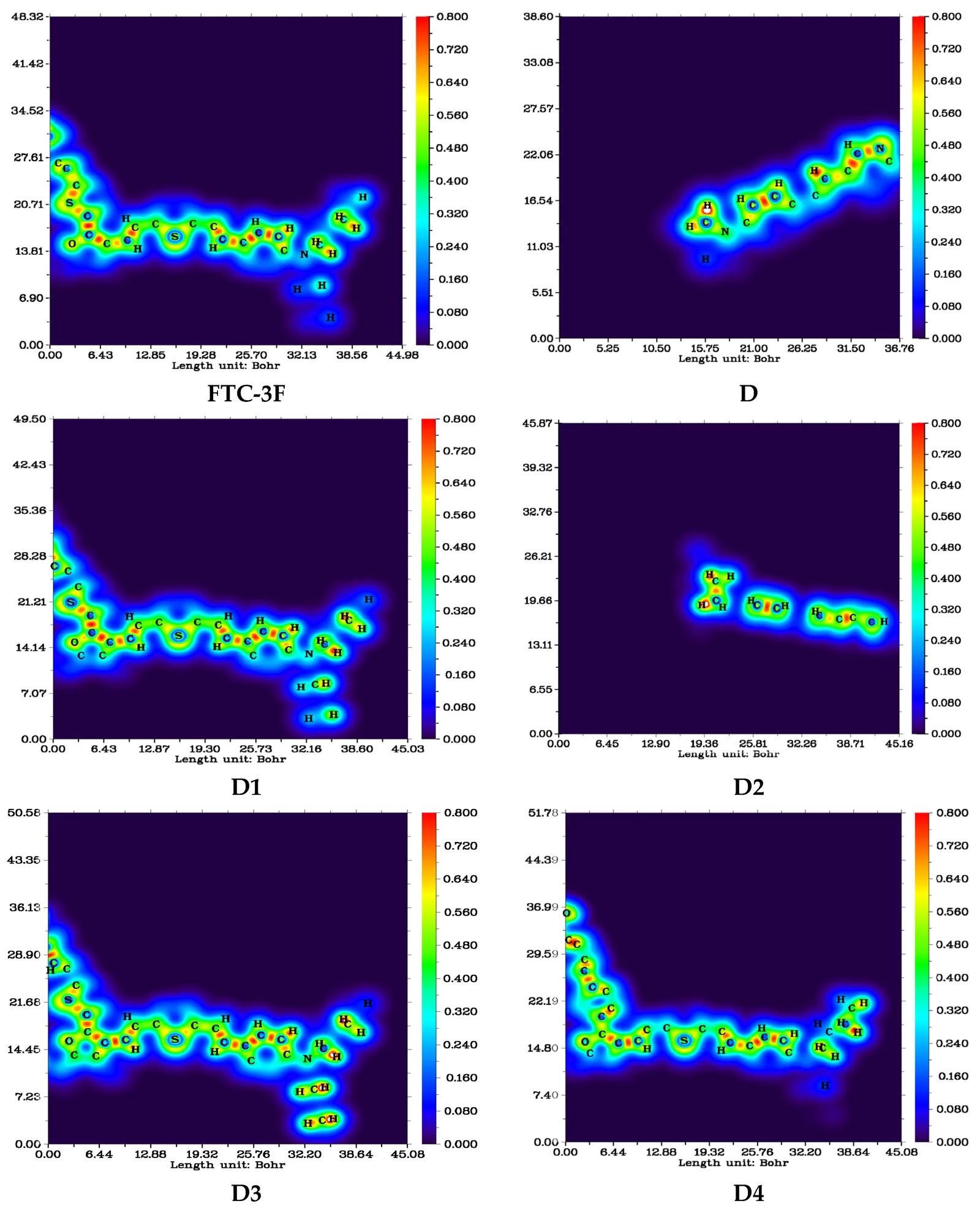
The LOL plots of all studied compounds.

The localized orbital locator (LOL) is a useful topological tool for studying electron localization within a given molecular system, as well as the bonding and charge delocalization. Unlike electron density methods, LOL can be derived from kinetic energy density and provides clear data on localized and delocalized electron regions, which are crucial for understanding nonlinear optical (NLO) behavior and intramolecular charge transfer (ICT) [41]. Figure 6 presents the two-dimensional (2D) LOL contour maps of FTC-3F and the proposed chromophores (D, D1–D4). The color gradient of the LOL maps is blue to red, with the extent of blue going to 0.8. Locations with an LOL value exceeding 0.5 (red) indicate high electron localization and strong bonding interactions, whereas those with a value less than 0.5 (blue) indicate low electron density. The presence of strong covalent bonds and localized σ-sigma bonding is evident in the red patches, with most occurring around hydrogen atoms and heteroatoms (N, O, and S). Conversely, blue-to-green regions of the π-conjugated structures of the chromophores reflect long-term delocalization of electrons along the molecular backbone. Interestingly, the proposed derivatives are more delocalized with the donor → acceptor structure; the D3 and D4 substituents with the strong electron-withdrawing substituents (NO_2_ and CF_3_) in particular show the expansion of ICT pathways and effective redistribution of charges that is needed to boost better electrical characteristics and superior NLO reaction. Overall, the LOL studies demonstrate enhanced nonlinear optical activities and theragnostic applications of the reported chromophores, confirming that electron delocalization is indeed enhanced through molecular engineering using spacer and acceptor modifications.

#### 2.1.7. TDM Analysis

**Figure 7:**
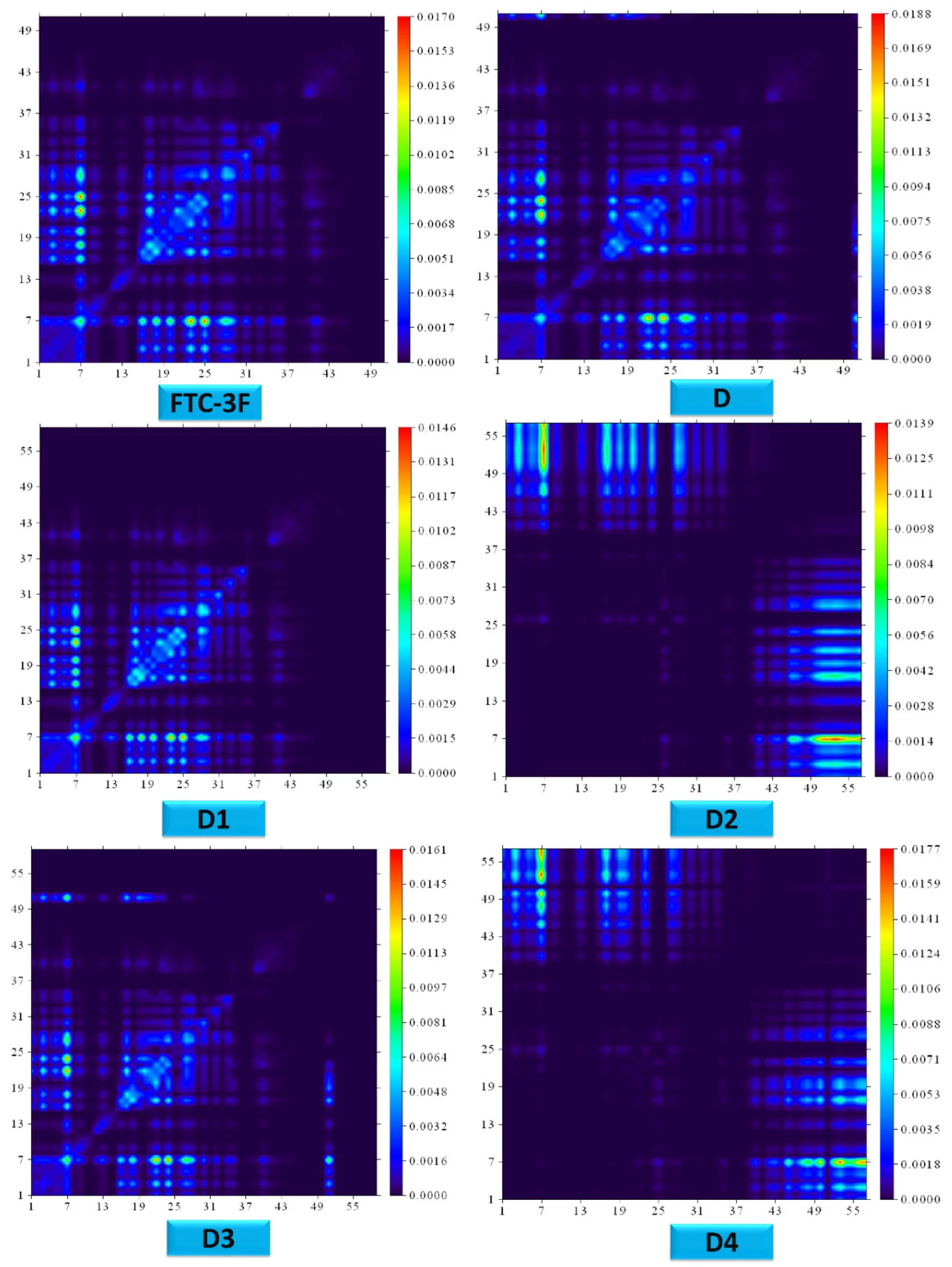
The TDM plots of all studied compounds.

A transition density matrix (TDM) was used to study the character of electronic excitations, pathways of charge transfer, and interactions between donors and acceptors in FTC-3F and the designed derivatives (D, D1–D4). It was studied at the aforementioned B3LYP/6-31G(d,p) level of theory. TDM maps can provide insight into the distribution of holes and electrons, as well as charge delocalization, at the time of excitation, with darker, stronger regions indicating a greater probability of transition [36,37]. In Figure 7, the parent molecule FTC-3F exhibits a relatively localized transition density in the molecular core, indicative of moderate intramolecular charge transfer (ICT). The molecules formed, on the other hand, have a higher degree of charge dispersion, and particularly in D3 and D4, a significant diagonal movement of the electron density toward the acceptor segments is observed. Powerful electron-withdrawing NO_2_ and CF_3_ groups promote efficient charge transfer between donor and acceptor units, resulting in long-range delocalization throughout the conjugated framework. Hence, it was observed that the successful transfer of electrons from the donor to the acceptor supports the successful modification strategy and leads to the best molecules for photonics and theragnostic applications.

Overall, from electronic properties through DFT computation, all the FMO, DOS, LOL, TDM, and TD-DFT UV–Vis computations show the same intramolecular charge transfer (ICT) process in the studied D–π–A chromophores. The calculations of FMO show that the HOMO is mainly localized on the donor fragment and the LUMO is mainly localized on the acceptor unit, which suggests the migration of an electron from the donor unit to the acceptor unit when excitation occurs. The dominance of donor-fragment contributions to the occupied states and of acceptor-fragment contributions to the unoccupied states is also evident in the DOS results. For D3 and D4, the LOL maps show that the delocalization of the π-electrons is increased along the π-conjugated backbone, which can lead to efficient electronic communication between the donor and the acceptor. Similarly, the TDM results validate effective charge transfer to the acceptor segment during excitation, consistent with a higher-ICT character. These electronic properties are reflected in the TD-DFT UV–Vis spectra, with successive bathochromic shifts in the designed derivatives attributable to reduced HOMO–LUMO energy gaps and increased charge transfer transitions. The overall analysis of these complementary methods leads to a coherent picture of their electronic behavior. It shows that spacer and acceptor modifications can enhance conjugation and efficient ICT, thereby improving the optical and nonlinear optical properties of the proposed chromophores. These descriptors are derived from the same electronic structure calculations but represent different perspectives on the same ICT mechanism and are not independent validations of the phenomenon.

### 2.2. Potential Application

In summary, the investigated compounds possess impressive biological and optoelectronic characteristics. Their ability to react and their strength are enhanced by their high dipole moments and low bandgap, which are attributed to decreased HOMO/LUMO energy levels. Also, molecular docking studies with certain target proteins yield favorable docking scores, suggesting potential involvement in biological systems. Moreover, these materials exhibit good absorption in the visible region, which is advantageous for photothermal applications.

### 2.3. Nonlinear Optical Properties

The nonlinear optical (NLO) response of FTC-3F and the designed chromophores (D, D1–D4) was studied by the calculation of the dipole moment (μ), the average polarizability (α), and the first hyperpolarizability (β) in a.u. in the gas phase and polar solvent (water) by the IEF-PCM model, as shown in Table 1 [42,43,44,45]. All of the proposed derivatives have enhanced NLO properties; the FTC-3F (reference molecule) shows 21.91 D (dipole moment), 840.52 (linear polarizability), and 1.21 × 10^5^ (first hyperpolarizability) in a.u. The hyperpolarizability increases in molecules having thiazole spacer D, showing 1.41 × 105 a.u., and 1.50 × 105 a.u. for D4. The first hyperpolarizability increases slightly, suggesting increased intramolecular charge transfer (ICT) due to structural alterations and extended π-conjugation. D4 exhibits strong electronic polarization, as evidenced by the highest values of μ (26.63 D), α (955.04 a.u.), and β (1.50 × 10^5^ a.u.) among all the designed compounds. In water, the solvent effect is noticed. FTC-3F has significantly higher values of dipole moment and linear polarizability (1355.67 a.u.) and first hyperpolarizability (5.63 × 10^5^ a.u.) compared to the gas phase. This tendency can be observed in the major growth of the values of first hyperpolarizability values of the produced chromophores: D4 (7.40 × 10^5^) > D3 (7.34 × 10^5^) > D2 (5.72 × 10^5^) > D1 (5.63 × 10^5^) > FTC-3F (5.63 × 10^5^) > D (1.41 × 10^5^) in a.u. The dipole moment is higher in the solvent phase (water), at 36.64 D for D4, thereby enhancing charge separation in a polar medium. The suggested chromophores exhibit moderate NLO responsiveness amplifications over FTC-3F in a non-polar solvent such as benzene, and D4 has the highest μ, α, and β; again, structural changes are largely responsible for enhancing NLO performance. Taken together, these results indicate that solvent polarity, strong electron-withdrawing substituents, and long-range (π-conjugated) character are synergistic factors that enhance NLO properties; thus, these chromophores are competitive choices in theragnostic and optoelectronic systems.

Table S5 (Supplementary Materials) summarizes the calculated values of the first hyperpolarizability tensor components (β_ZZZ_). When the structure of the reference molecule is altered, a significant improvement in NLO response is realized. FTC-3F shows the lowest value of β_HRS_ (2.97 × 10^4^ a.u.), and the designed derivatives reflectively increase in response in the following order: FTC-3F < D < D1 < D2 < D3 < D4. D4 is the most hyperpolarizable system (hyperpolarizability of 5.11 × 10^4^ a.u.), followed by D3 (hyperpolarizability of 4.68 × 10^4^ a.u.), which is to be expected because the inclusion of spacers based on thiazoles and more potent acceptor fragments is a significant factor driving the increase in second-order NLO activity. The thiophene-based derivatives (D1 and D2) also exhibited better responses than FTC-3F, but their NLO amplitudes were lower than those of D3 and D4. This improvement can be explained by the synergistic impact of longer π-conjugation, stronger donor–acceptor interactions, and enhanced intramolecular charge transfer (ICT). The more nitrogen, sulfur, oxygen, and fluorine in the acceptor fragment, the more electronic softness and the greater ability to displace charges in the presence of an external electric field the designed derivatives exhibit. That the β_ZZZ_ is much stronger than 2nd-ranking tensors such as β_zxx_ indicates that the above chromophores are rather one-dimensional push–pull NLO systems, with polarization predominantly along the molecular axis. The values of the DR also rise considerably between FTC-3F and D4, which shows the growing anisotropic and directional character of the nonlinear response. From these findings, it was concluded that D3 and D4 are the best candidates for second-order NLO, consistent with their increased charge transfer and enhanced electronic delocalization. The static NLO parameters were also computed using the diffuse-function basis sets 6-31+G(d,p) and 6-31++G(d,p) to investigate the effect of diffuse functions on the computed properties (Table S6). The calculated dipole moment (μ), average polarizability (α), and first hyperpolarizability (β) values vary only slightly from those calculated with the 6-31G (d, p) basis set, and the relative performance of the designed chromophores does not change, with D4 > D3 > D > D2 > D1 > FTC-3F (hyperpolarizability). The results shown above confirm that the obtained enhancement of the NLO response is stable under the addition of diffuse functions and further validate the reliability of the present comparative analysis. The calculated value of first hyperpolarizability (β) was then compared with the values obtained with the exchange-correlation functional CAM-B3LYP and basis sets 6-31+G (d, p) and 6-31++G (d, p) for the reference compound FTC-3F and for the representative derivatives D and D4 to check the sensitivity of the results to the exchange-correlation functional and to the basis sets employed. The corresponding results, together with the original B3LYP/6-31G (d, p) values, are summarized in Table S5 (Supplementary Materials). The calculated hyperpolarizabilities at B3LYP are in reasonable agreement with those calculated at B3LYP/6-31G (d, p), and the difference is due to the sensitivity of calculated hyperpolarizabilities of D–π–A systems to the treatment of long-range exchange. At the CAM-B3LYP/6-31+G (d, p) level, the calculated β values for FTC-3F, D, and D4 are 1.12 × 10^5^, 1.24 × 10^5^, and 1.66 × 10^5^ a.u., respectively, whereas the corresponding values at the CAM-B3LYP/6-31++G (d, p) level are 1.12 × 10^5^, 1.24 × 10^5^, and 1.65 × 10^5^ a.u. These values can be compared with 1.21 × 10^5^, 1.41 × 10^5^, and 1.50 × 10^5^ a.u. obtained using B3LYP/6-31G (d, p) for FTC-3F, D, and D4, respectively. Both functionals have the same ordering, but not in absolute terms, D4 > D > FTC-3F. Furthermore, the very small difference in the calculated β value for these representative systems between the diffuse basis sets 6-31+G (d, p) and 6-31++G (d, p) suggests that the diffuse functions have little influence on the NLO results. The results suggest that the absolute value of the calculated β is sensitive to the exchange-correlation functional; however, the relative trends in β across the different chromophores are the same. This agreement strengthens the comparative NLO analysis but prevents overinterpreting the absolute B3LYP values.

**Figure 8:**
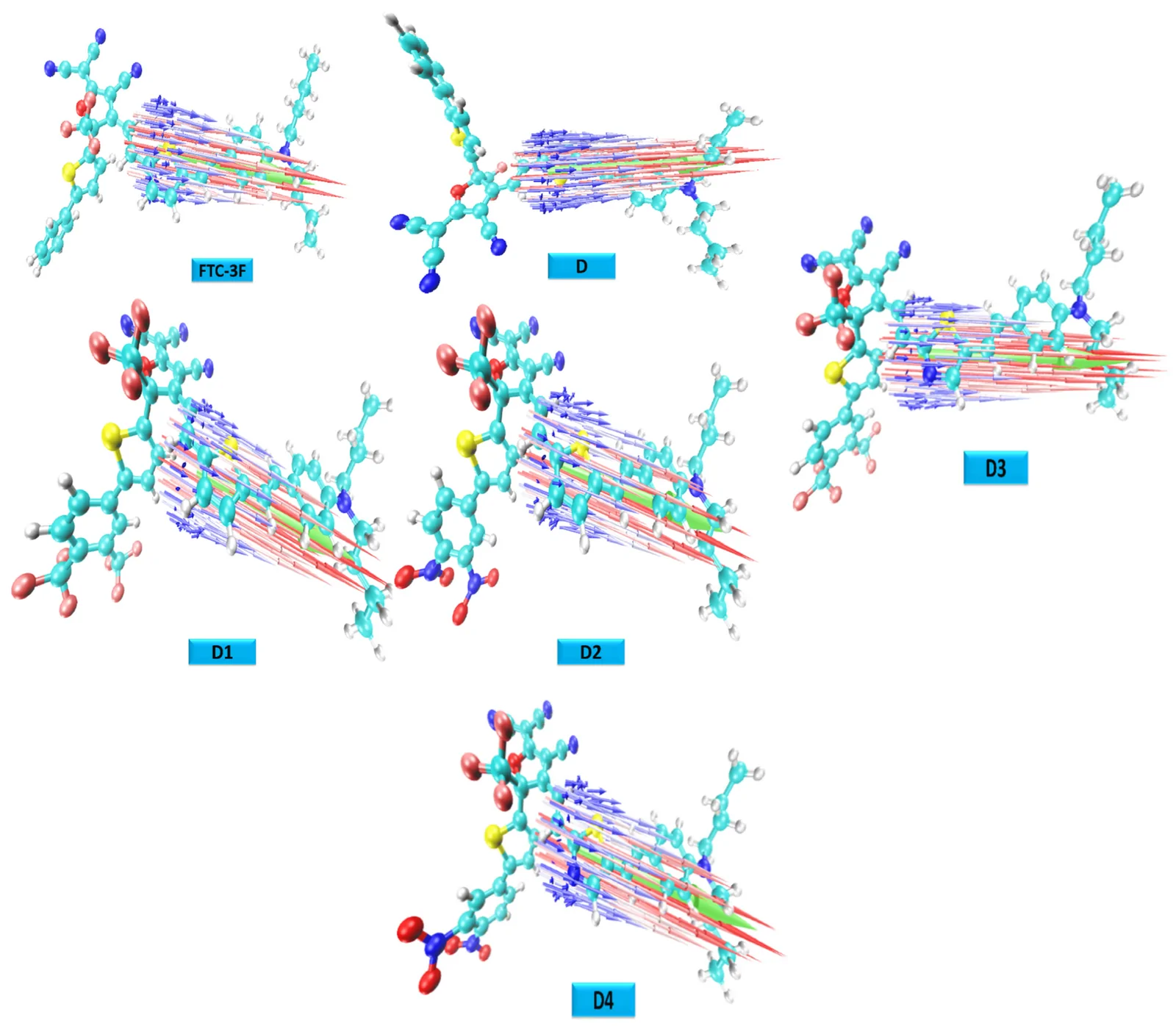
The first hyperpolarizability tensors of FTC-3F and its designed analogs (D–D4), which show the directional distribution and anisotropy of the second-order nonlinear optical response, represented in unit sphere form calculated at the TDDFT/B3LYP level of approximation (USR factor of 0.0001). The red and blue coloring denotes the sign (positive and negative, respectively) of the projected first hyperpolarizability component along a given direction. In contrast, the magnitude of the response in that direction is represented by the radial distance of the surface from the origin.

To further elucidate the spatial orientation and anisotropy of the nonlinear response, a unit sphere representation (USR) of the first hyperpolarizability tensors was examined, as depicted in Figure 8. The USR plots provide a graphical representation of the direction and magnitude of the induced second-order dipole moment in response to an applied electric field [46]. In the case of the reference molecule FTC-3F, the USR pattern is neither very strong nor elongated, indicating that the nonlinear response is relatively small and poorly oriented. This coincides with its low β_HRS_ value and poor charge transfer properties. The USR distribution decreases in size and becomes increasingly directional along the molecular backbone upon structural modification. These high ICT characteristics make it favorable for their potential application in multifunctional optoelectronic and photothermal systems.

### 2.4. Frequency-Dependent NLO Behavior

Further investigation of the dynamic nonlinear optical response of the studied chromophores was performed by calculating the frequency-dependent first hyperpolarizability at 532, 1064, and 1907.21 nm for FTC-3F and its designed derivatives (D–D4) [47]. Table 2 shows the calculated electro-optical Pockels effect (EOPE), β (−ω, ω, 0), and second harmonic generation (SHG), β (−2ω, ω, ω) [48]. The findings show that the designed derivatives exhibit a pronounced frequency-dependent NLO response, and their EOPE and SHG values are much higher than those of the reference molecule FTC-3F. The EOPE values decrease in the following order at 1064 nm: FTC-3F (−1.01 × 10^1^) < D (1.29 × 10^2^) < D1 (1.92 × 10^5^) < D2 (2.01 × 10^5^) < D3 (2.56 × 10^5^) < D4 (2.64 × 10^5^) in a.u. On the other hand, SHG response shows slight variation relative to EOPE at 1064 nm in the following manner: D1 > D2 > D4 > D3 > FTC-3F > D. At 532 nm, the EOPE increases for D1 (1.61 × 107 a.u.), and SHG is highest for D4 (1.17 × 10^6^ a.u.). Both responses are lowest for D (EOPE = −7.02 × 10^7^ a.u. and SHG = −1.03 × 10^6^ a.u.). The EOPE and SHG properties were also investigated at 1907.21 nm to further examine the wavelength dependence of the nonlinear optical response. As stated in the summary of Table 2, the calculated EOPE values increase in the order D1 (9.56 × 10^4^ a.u.) < D2 (9.92 × 10^4^ a.u.) < D (1.09 × 10^5^ a.u.) < D3 (1.14 × 10^5^ a.u.) < D4 (1.17 × 10^5^ a.u.), whereas the reference FTC-3F shows an EOPE value of 9.13 × 10^4^ a.u. Similarly, the SHG response follows the trend FTC-3F (1.66 × 10^5^ a.u.) < D1 (1.81 × 10^5^ a.u.) < D2 (1.89 × 10^5^ a.u.) < D (2.17 × 10^5^ a.u.) < D3 (2.40 × 10^5^ a.u.) < D4 (2.47 × 10^5^ a.u.). Of all designed derivatives, D4 shows the best EOPE and SHG responses, which have resulted from the beneficial effect of stronger donor–acceptor interactions, extended π-conjugation, and enhanced intramolecular charge transfer on the dynamic nonlinear optical properties.

These findings show that the NLO response of the studied systems is strongly dependent on the applied optical frequency and is significantly enhanced by molecular modification. Structurally, the enhanced dynamic hyperpolarizability of the derivatives obtained is due to the superior interaction of the donors and acceptors, increased π-elongation, and better intramolecular charge transfer (ICT). The derivatives of thiazole (D3 and D4) and thiophene (D1 and D2) exhibit the most balanced and pronounced dynamic NLO behavior, with D4 and D1, respectively, at 532 and 1064 nm. Of all systems, D4 has the most pronounced total frequency-dependent NLO response, whereas D1 exhibits the largest resonance-assisted enhancement under certain conditions. On the whole, the frequency-dependent data agree with the earlier reported static hyperpolarizability and PET measurements, indicating that π-spacer engineering and acceptor control are successful methods for enhancing the dynamic NLO characteristics of the examined chromophores. These conclusions identify D3 and D4 as the most promising for electro-optic and harmonic generation, as well as for higher photonic applications.

### 2.5. Biological Potential Through Molecular Docking

**Figure 9:**
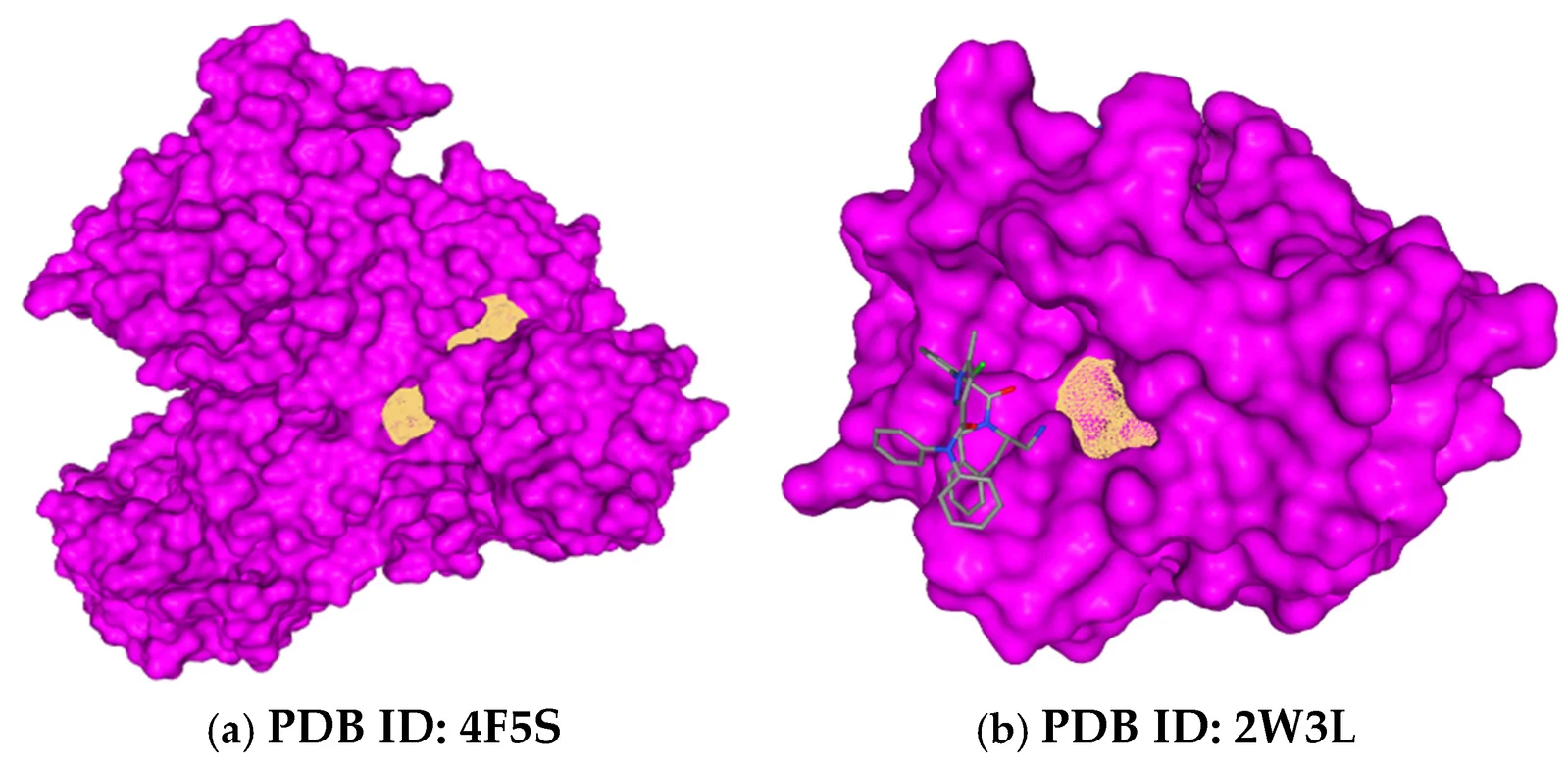
Identification of the active site by using DoGSiteScorer.

Biological targets were selected for their vital roles in transport, bioavailability, and apoptosis in photodynamic/photothermal therapeutic systems. Bovine serum albumin (PDB ID: 4F5S) was used to study transport and distribution, whereas Bcl-2 (PDB ID: 2W3L) was used to study apoptosis-related interactions. The Protein Data Bank was used to obtain the three-dimensional crystal structure of the two proteins. A single experimentally reported compound (FTC-3F) served as a reference, and the designed derivatives (D, D1–D4) were optimized on Density Functional Theory before docking. Active binding sites were identified, and docking was performed to assess binding strength and interaction profiles (Figure 9). The best conformations after docking were selected based on the docking score (S) and RMSD values (Table S7). The findings indicated that, in their top-ranked docking poses, the compounds occupied the hydrophobic pockets of both proteins, facilitated by hydrogen bonding, halogen interactions, and extensive π-interactions.

### 2.6. Visualization of Interaction with Serum and Apoptotic Protein

**Figure 10:**
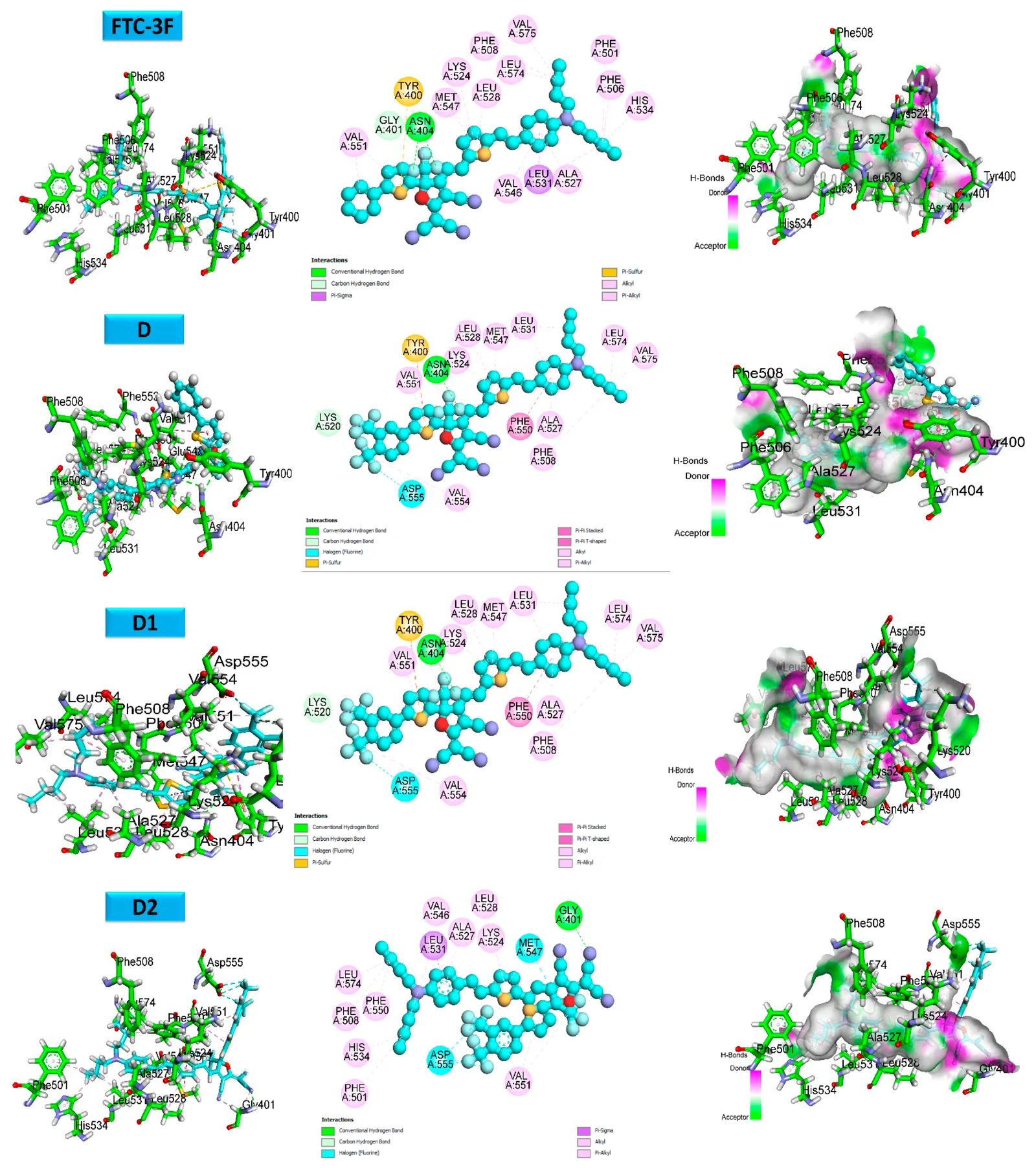

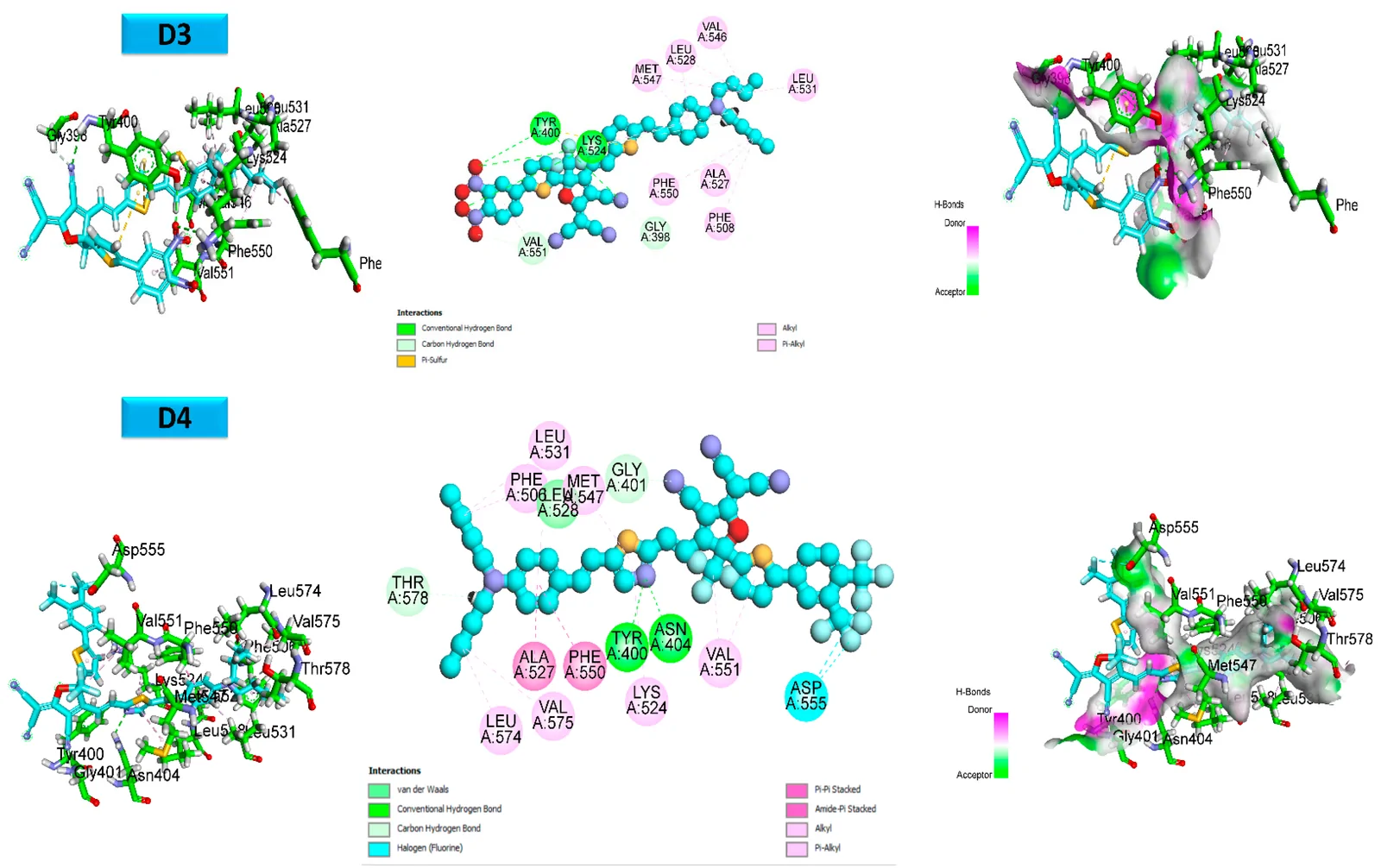
FTC-3F, D, D1, D2, D3, and D4 binding in the active site of **4F5S** protein. The 2D interaction diagrams (**left**) reveal the major contacts, such as hydrogen bonding and polar and hydrophobic interactions with other amino acid residues. The 3D models (**right**) characterize the spatial orientation and proper fit of FTC-3F, D, D1, D2, D3, and D4 within the active binding pocket, as well as the hydrogen-bond donor and acceptor surfaces, to validate stable and preferred binding poses. Table S8 reports the type of interactions and distances of the ligands to the interacting amino acid residues.

The order of Vina docking scores (S, kcal/mol) in the case of bovine serum albumin docking is as follows: D (−7.05) > D2 (−8.99) > D1 (−9.45) > D3 (−9.52) > D4 (−9.64) > FTC−3F (−10.47). The most favorable (most negative) docking score is observed with the reference compound FTC-3F, which means that there is a strong interaction with the transport protein. The docking of the protein and the designed ligands is shown in Figure 10 and Figures S1 and S2 (Supplementary Materials). At the interaction level, FTC-3F forms strong conventional hydrogen bonds with ASN404 and carbon–hydrogen bonds with GLY401. The interactions of π-alkyl groups with the residues PHE506, PHE508, VAL546, and MET547, and of π-sulfur with TYR400, are found to stabilize the complex. The fact that several hydrophobic residues are present (LEU, VAL, MET) suggests that it is well accommodated in the hydrophobic binding pocket. It was also observed that D shows the same pattern but a less favorable (less negative) docking score. D1 and D4 have a strong interaction network, including hydrogen bonding with TYR400 and ASN404, π–π stacking (PHE550), and amide–π stacking (ALA527). Compound D2 has fewer hydrogen bonds and strong halogen interactions (ASP555, MET547), whereas D3 has several hydrogen bonds with TYR400 and LYS524, consistent with a polar interaction network in this docking pose. In general, the favorable affinity of FTC-3F and the similar efficiencies of D1 and D4 indicate that the developed compounds preserve the high-quality transport properties required for biological distribution.

In the case of Bcl-2 docking, the order of Vina docking scores is as follows (S, kcal/mol): D2 (−8.54) ≈ D (−8.50) > FTC−3F (−8.40) > D4 (−8.01) > D1 (−7.81) > D3 (−7.05). Surprisingly, the best docking score is observed for the designed compound D2, which outperforms the reference compound. FTC-3F has favorable predicted interactions via hydrogen bonding with ARG88, a GLU95–π-anion interaction, and a PHE63 π–π T-shaped interaction. These predicted interactions are consistent with binding in the active site within this docking model. Compound D exhibits a broad spectrum of halogen bonding with ARG105, GLU95, and ASP99, together with hydrogen bonding and π-alkyl interactions, creating a robust interaction network. Compound D2 shows notable hydrogen bonds with ARG105, strong π-donor hydrogen bonding (ARG66), and π–π interactions with PHE63. This rich network of interactions justifies its greatest docking score. Compound D3 has fewer interactions and weaker binding, whereas D4 has balanced interactions with hydrogen bonding, π-sigma, and amide–π stacking (GLU73). In general, the molecular docking findings suggest an association between docking score and the suggested therapeutic use of the researched chromophores, indicating that ligand–protein interactions are key factors in their biological performance. The compounds that had higher binding scores with bovine serum albumin and Bcl-2 had greater predicted interaction stability, which is consistent with, but does not by itself establish, more favorable transport behavior and apoptosis targeting, a crucial aspect of photothermal therapeutic use. The analysis of interactions showed that a wide variety of non-covalent interactions, including traditional hydrogen bonding, carbon–hydrogen interactions, π-sigma, π-alkyl, alkyl, π–π stacking, π-anion, and halogen bonding with the major active-site residues, determine the stability of the ligand–protein complexes. These interactions are consistent with the formation of stable, energetically favorable complexes in the protein binding cavities within the docking protocol used. The extended π-conjugation and donor–acceptor architecture of the designed chromophores play a key role in enhancing their binding ability by strengthening electronic interactions with biological targets.

### 2.7. Molecular Descriptors

Molecular descriptors (MD) analysis is performed to develop quantitative correlations between biological or physicochemical properties and molecular structure [49]. It assists in anticipating the actions of novel compounds and in the rational development of more effective molecules. SMILES notation was used to construct and describe the molecular structures of the reference chromophore FTC-3F and its designed derivatives (D, D2, and D4) and convert them into three-dimensional geometries. The optimized structures were then examined using the RDKit cheminformatics toolkit to generate molecular descriptors, available online at https://www.rdkit.org/.

**Figure 11:**
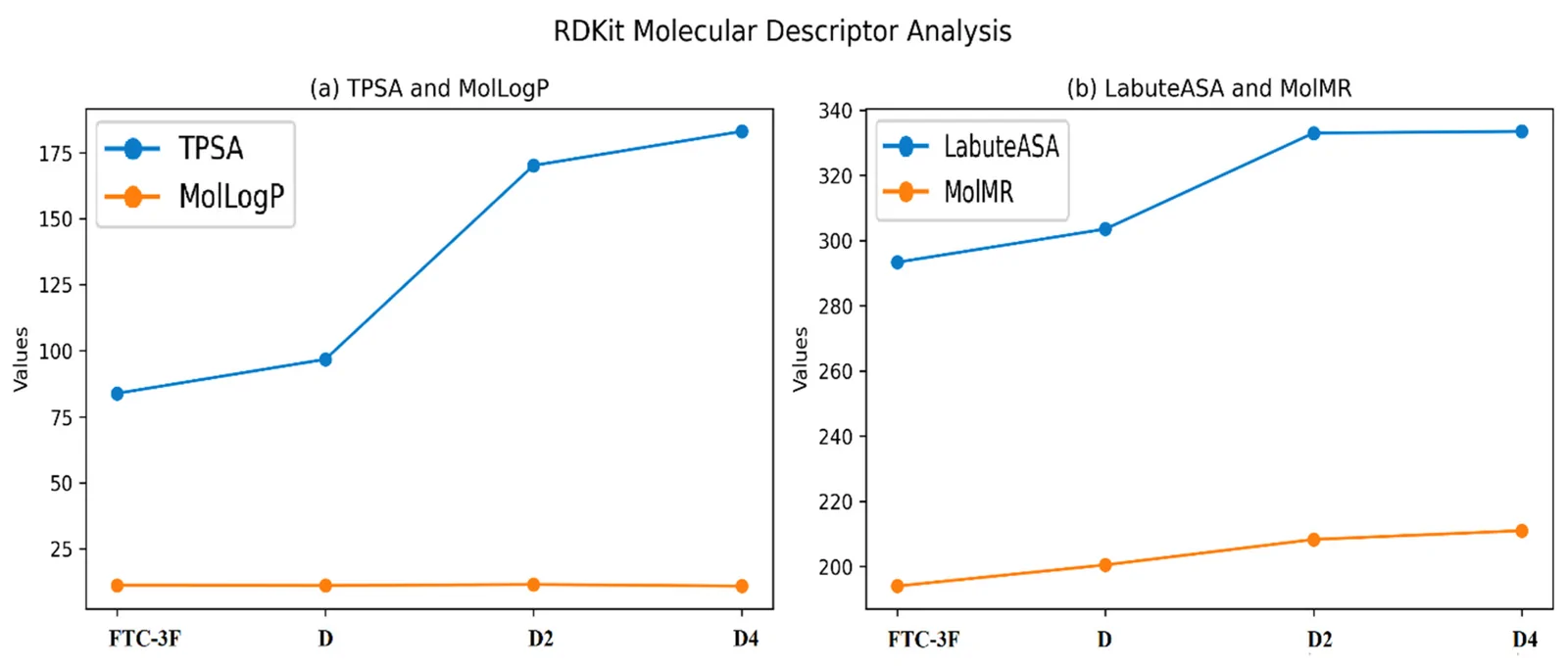
The 3D molecular descriptors in graphical form.

In the research, 3D-relevant and surface-dependent descriptors were weighted, including solvent-accessible surface area (LabuteASA), topological polar surface area (TPSA), molar refractivity (MolMR), lipophilicity (MolLogP), and descriptor families based on partial charge distribution (PEOE_VSA and EState_VSA). The combination of these descriptors provides a complete picture of molecular size, shape, polarity, electronic distribution, and surface accessibility, which are key parameters in the analysis of structure–property relationships. No regression-based model was built, unlike traditional QSAR model development. Rather, a direct, descriptive-based comparative analysis was used to assess the impact of structural changes. This method reduces statistical bias and enables a better understanding of the effects of spacer engineering and acceptor substitution on the physicochemical characteristics of the chromophores. The extensive molecular descriptor analysis was performed using RDKit to assess structural changes in the reference chromophore, FTC-3F, and its derivatives (D, D2, and D4). The calculated descriptors, physicochemical, topological, and surface-related parameters, help evaluate changes in molecular size, polarity, lipophilicity, and structural complexity. Replacement of the thiophene unit in FTC-3F with a thiazole moiety in D gave spacer engineering, which increased the molecular weight (721.87 vs. 696.86 g mol^−1^), solvent-accessible surface area (LabuteASA: 303.55 vs. 293.35 A2), and topological polar surface area (TPSA). This change also increased the BertzCT index (2063.65 vs. 1896.99), indicating greater topological complexity, while minimally decreasing hydrophobicity (MolLogP: 11.071 vs. 11.208). These effects were greatly enhanced by further acceptor engineering via nitro replacement in D2 and D4 to produce higher molecular weights (~810812 g mol^−1^), larger surface areas (~333 A^2^), and significantly increased TPSA values (170.12 and 183.01 A^2^, respectively), as well as an increased number of hydrogen bond acceptors and heteroatom density. Increased polarizability was also indicated by higher structural complexity (BertzCT > 2400) and higher molar refractivity (>211) of these derivatives. Although the polarity increased, all compounds were very lipophilic (MolLogP > 10); nevertheless, the balance was the most favorable (the lowest MolLogP, 10.888) and the TPSA was the largest, indicating better physicochemical properties in D4. Also, there was a gradual increase in molecular size, steric bulk, and flexibility in the series, as evidenced by increases in LabuteASA, the number of heavy atoms and rotatable bonds, and by variations in the Kappa and Chi indices, which indicate changes in molecular geometry. The overall trend of the descriptors is FTC-3F < D < D2 < D4, which indicates that collective spacer and acceptor engineering is effective in optimizing molecular properties and yields the most comprehensive descriptive profile, with D4 being the most balanced candidate. In addition to RDKit-based descriptors, frontier molecular orbital (FMO)-based descriptors obtained from Gaussian calculations also support the trends observed in the descriptor analysis. As shown in Table S9 (Supplementary Materials), it can be seen that ionization potential (IP) and electron affinity (EA) steadily increase as FTC-3F transforms to D4 (IP: 5.209 to 5.568 eV; EA: 3.252 to 3.867 eV), which is an indication of electronic stability and enhanced electron-withdrawing capacity of structural modification. Thus, the electronegativity (χ) grows systematically (4.230 4.718 eV), indicating a better capacity of the altered chromophores to attract electrons. In the meantime, there is a slight downward trend in chemical hardness (η) (0.978 0.851 eV) and an increase in softness (σ: 0.511 0.588 eV^−1^), indicating that the designed derivatives become more polarizable and chemically reactive. Chemical potential (μ) is more negative (−4.230–4.718 eV), which again proves the increase in stability of the electron cloud in the modified systems (Figure 11). It is interesting to note that the electrophilicity index (ω) goes up substantially in the series (9.147–13.083 eV) with the nitro-substituted derivatives being the better electron acceptors. These findings are consistent with the RDKit descriptor analysis, which indicates that spacer and acceptor engineering can alter molecular size and polarity, as well as electronic properties, with D4 showing the most favorable balance of reactivity, stability, and electrophilic character among the analyzed chromophores. In general, the theoretically designed NIR-absorbing chromophores have excellent potential as multifunctional NLO materials for future photothermal therapeutic applications, owing to their combination of efficient light-to-heat conversion and favorable protein interaction properties.

## 3. Materials and Methods

### 3.1. Quantum Chemical Method

The Gaussian 16 [50] program was employed to perform quantum-chemical calculations (DFT and TD-DFT) (15) on the designed chromophores at the B3LYP/6-31G(d,p) level (16) using default parameters: temperature = 298.15 K and pressure = 1 atm. The development of novel NLO materials (FTC-3F, D, and D1–D4) with a D–π–A configuration was achieved through spacer and acceptor modifications, owing to their superior electron transfer and charge mobility compared to conventional acceptors and spacers [32], as well as the enhanced donating ability of the donor moiety, N, N-diphenylnaphthalen-1-amine. After verifying that the frequency measurements contained no negative imaginary values, all ChemDraw structures were optimized to their ground-state energy minima [51]. The structures of our reference compound (FTC-3F) were visualized and analyzed utilizing GaussView 5.0 [52]. The λ_max_ values derived from UV–Vis using B3LYP were the most relevant to their experimental counterparts compared to CAM-B3LYP (see supporting Table S3a,b). The TDM, LOL, and ELF investigations were conducted using Multiwfn 3.7 [53] to examine the charge mobility of the proposed compounds. We employed the Multiwfn and VMD 1.9.4 software to obtain diagrams and energies of frontier molecular orbitals (FMOs) [53]. The spectral diagram was generated in Origin 8.0 [54], and the ultraviolet–visible (UV–Vis) analysis was performed with GaussSum 3.0 [55]. Moreover, PyMOlyze 2.0 [55] was employed to generate density of states plots. The influence of the solvent on nonlinear optical properties was analyzed using the IEFPCM model. To check the solvent effect for enhancing the absorption maximum (near-IR response theoretically) in FTC-3F as reported in the literature [28], the CPCM model is used with solvent tetrahydrofuran (ε = 7.43), THF at B3LYP/6-31G (d, p), and the maximum absorption calculated is 756.97, which is very close to the experimental wavelength 728 nm in THF solvent. Moreover, cc-pVDZ/cc-pVTZ basis set calculations vs. 6-31G (d, p) show 549.12, 560.19 nm vs. 653.339 nm, so this further supports that the B3LYP/6-31G (d, p) level of theory is the best functional for this study.

Utilizing the B3LYP/6-31G (d, p) level of theory and Equations (1)–(4), the nonlinear optical properties, including linear polarizability (<α>) [56], total nonlinear hyperpolarizability (β_tot_), and hyper-Rayleigh scattering (β_HRS_) [57], were computed, in addition to the dipole moment (µ_tot_) [58].

The frequency-dependent NLO response was evaluated by calculating the electro-optic Pockels effect (EOPE), β (−ω, ω, 0), and second-harmonic generation (SHG), β (−2ω, ω, ω), at laser wavelengths of 1906 nm (ω = 0.02389 a.u.), 1064 nm (ω = 0.0428 a.u.), and 532 nm (ω = 0.0856 a.u.).

The hyper-Rayleigh scattering (β_HRS_) is calculated as follows:

(1) $$\beta_{\mathrm{HRS}}\;(-2\omega ,\;\omega ,\;\omega )\;=\;\sqrt{\langle \beta_{zzz}^{2}\rangle +\langle \beta_{xzz}^{2}\rangle }$$

The following equation determines EOPE values:

β_i_ = β_iii_ (−ω, ω, 0) + β_ijj_ (-ω, ω, 0) + β_ikk_ (−ω, ω, 0) (2)

SHG calculation is performed by following the mentioned equation:

β_i_ = β_iii_ (−2ω, ω, ω) + β_ijj_ (−2ω, ω, ω) + β_ikk_ (−2ω, ω, ω) (3)

The depolarization ratio is calculated by:

(4) $$\mathrm{DR}\;=\;\frac{\langle \beta_{zzz}^{2}\rangle }{\langle \beta_{xzz}^{2}\rangle }$$

### 3.2. Molecular Docking

To confirm the valid binding interactions of the designed ligand D4 (best-performing compound) in comparison with the reference photosensitizer FTC-3F with the chosen protein targets, bovine serum albumin (BSA) and Bcl-2, molecular docking was performed. ChemDraw Professional 16.0 was used to draw the 2D chemical structures of FTC-3F and D4 with appropriate orientations, and the resulting structures were then imported into Chem3D 16.0 to minimize energy and obtain stable, low-energy conformations. The 3D crystal structures of the target proteins were downloaded from the RCSB Protein Data Bank (PDB) and analyzed for docking (available online: https://www.rcsb.org/ [59]). Crystal structures of BSA (PDB ID: 4F5S; resolution: 2.47 Å) and Bcl-2 (PDB ID: 2W3L; resolution: 2.10 Å) were obtained, and BSA was chosen to study transport and bioavailability-mediated binding of the photosensitizer, whereas Bcl-2 was chosen to study apoptosis-related interactions that are associated with PDT response. Due to docking, the binding pockets/active sites of both proteins were predicted by DoGSiteScorer (accessed on 20 February 2026) before docking. To make receptors ready to dock, co-crystallized ligands (where present) were removed, solvent molecules were removed, residues or atoms were replaced as needed, polar hydrogens were added, and the correct charges were assigned. The minimized ligands and resulting receptors were converted to PDBQT format. The docking grid box was centered at x = 18.121, y = 33.916, and z = 100.244 with a value of exhaustiveness of 8 and dimensions of x = 92, y = 66, z = 66 in the case of 4F5S and, for 2W3L, the grid box was centered at x = 41.153, y = 36.479, and z = −2.664 with a value of exhaustiveness of 8 and dimensions of x = 74, y = 82, z = 76. All these molecular docking analyses are performed using AutoDock Vina 1.1.2 [60]. A maximum of five ligand conformers can be generated during the docking process, and the most favorable docking conformation was decided by selecting a posture that has the lowest estimated binding free energy. The resulting complexes were studied for protein–ligand interactions using PyMOL 2.5.8 [61] and Discovery Studio Visualizer (BIOVIA DS 2024 version) [62].

### 3.3. Validation of Protein

**Figure 12:**
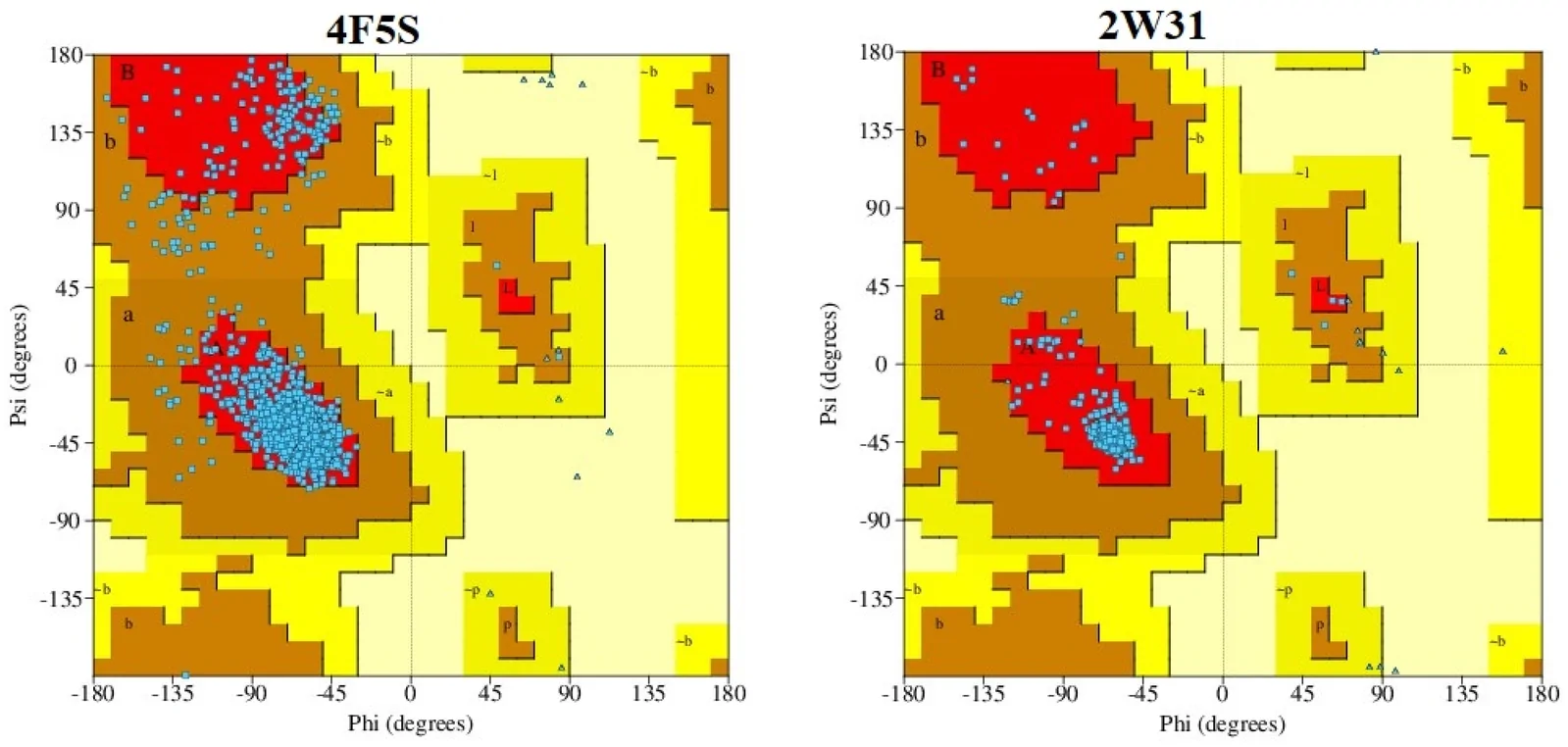
The Ramachandran plots of proteins.

The quality of the selected protein structures in terms of stereochemistry was assessed by analyzing Ramachandran plots generated by PROCHECK (accessed on 20 February 2026) (Figure 12). In the case of protein 4F5S, 91.9% of residues were found in the most preferred regions, and 8.1% were found in additionally permitted regions. No residues were found in generously permitted or dispersed regions. In the same way, Bcl-2 protein (PDB ID: 2W31) had 96.0% residues in most preferred regions, 4.0% in additionally allowed regions, and 0.0% in both generously allowed and disallowed regions. The negligible residues in the prohibited areas and the high ratio of residues in preferred areas indicate the high stereochemical and structural integrity of both proteins, which justifies their suitability for the molecular docking investigation.

## 4. Conclusions

This research involved designing several D–π–A compounds (D, D1–D4) and embedding intrinsic electron acceptor moieties and spacer (B) units to maximize nonlinear optical (NLO) and optoelectronic reactivity. All electronic, NLO, and biological interaction results are theoretical predictions from a single level of theory (B3LYP/6-31G (d, p)) with implicit solvation and in silico docking. The quantum chemical analyses indicated that all compounds generally have efficient intramolecular charge transfer, yet with the lowest HOMO–LUMO gap (1.701–1.925 eV) and largest first hyperpolarizability (1.50 × 10^5^–1.21 × 10^5^) and dipole moment (26.63–21.20 D). Strong charge separation and effective electronic transitions across the series were consistently verified using PET, DOS, LOL, USR plots, and TDM analyses. A red-shifted, higher-intensity NIR absorption (653.34–715.43 nm) is visible throughout the series in the absorption spectra, suggesting favorable binding conformations and consistent with potential transport and apoptotic-targeting behavior, which would require experimental confirmation. Molecular docking between bovine serum albumin (PDB ID: 4F5S) and Bcl-2 (PDB ID: 2W3L) was investigated, indicating a promising capability for transporting and targeting apoptotic cells. These theoretical predictions open the door for further experimental research to design and validate such chromophores as potential candidates for nanomedicine. Among the designed chromophores, D4 is the ideal D–π–A. It offers an effective combination of an outstanding NLO response, charge transfer, NIR functionality, and biological compatibility, making it a potential candidate for multifunctional photonic and photothermal applications. Further research will involve experimental validation of these computational predictions to realize their full potential in state-of-the-art optical and therapeutic systems.

## Figures and Tables

**Table 1:** The non-linear optical parameters were calculated in a gas and a highly polar solvent (water).

Gas Phase			
Molecule	Dipole Moment	Polarizability (α)	Hyperpolarizability (β)
FTC-3F	21.913	840.522	1.21 × 10^5^
D	21.201	845.031	1.41 × 10^5^
D1	23.700	920.528	1.26 × 10^5^
D2	23.400	944.167	1.30 × 10^5^
D3	23.353	933.063	1.46 × 10^5^
D4	26.627	955.039	1.50 × 10^5^
**Polar Solvent (Water)**			
FTC-3F	30.808	1355.674	5.63 × 10^5^
D	21.201	845.031	1.41 × 10^5^
D1	33.154	1097.751	5.63 × 10^5^
D2	32.274	1139.197	5.72 × 10^5^
D3	32.663	1177.625	7.34 × 10^5^
D4	36.645	1215.312	7.40 × 10^5^
**Non-Polar Solvent (Benzene)**			
FTC-3F	25.879	1035.723	2.46 × 10^5^
D	25.002	1054.838	2.96 × 10^5^
D1	27.901	1137.852	2.51 × 10^5^
D2	27.402	1170.117	2.57 × 10^5^
D3	27.476	1171.189	3.01 × 10^5^
D4	31.061	1200.808	3.06 × 10^5^

**Table 2:** The electro-optical Pockels effect (EOPE) and second harmonic generation (SHG) at 532, 1907.21, and 1064 nm.

	At 1064 nm		At 1907.21 nm		At 532 nm	
Molecules	EOPE	SHG	EOPE	SHG	EOPE	SHG
FTC-3F	−1.01 × 10^1^	2.41 × 10^2^	9.13 × 10^4^	1.66 × 10^5^	6.83 × 10^3^	−1.04 × 10^3^
D	1.29 × 10^2^	−1.19 × 10^4^	1.09 × 10^5^	2.17 × 10^5^	−7.02 × 10^7^	−1.03 × 10^6^
D1	1.92 × 10^5^	4.06 × 10^5^	9.56 × 10^4^	1.81 × 10^5^	1.61× 10^7^	4.12 × 10^4^
D2	2.01 × 10^5^	2.87 × 10^5^	9.92 × 10^4^	1.89 × 10^5^	2.52 × 10^6^	6.50 × 10^4^
D3	2.56 × 10^5^	2.59 × 10^5^	1.14 × 10^5^	2.40 × 10^5^	3.54 × 10^5^	9.46 × 10^5^
D4	2.64 × 10^5^	2.80 × 10^5^	1.17× 10^5^	2.47 × 10^5^	1.09 × 10^7^	1.17 × 10^6^

## Data Availability

Publicly available protein structure data were obtained from the RCSB Protein Data Bank (PDB) under the accession IDs 4F5S and 2W3L. All other data generated or analyzed during this study are included within the article and its Supplementary Materials.

## Supplementary Materials

The following supporting information can be downloaded at: https://www.mdpi.com/article/10.3390/ijms27167468/s1.
