# Supplementary material for: Quantum Chemical Tailoring of Donor–Acceptor Organic Nanomedicines for Nonlinear Optical Performance and Theragnostic Applications: Molecular Descriptors, In Silico, and Ab Initio Investigations
Source: Int J Mol Sci. 2026 Aug 20;27(16):7468. doi: 10.3390/ijms27167468 (PMC13513749; doi:10.3390/ijms27167468)
Supplement: Supplementary file 1 [file ijms-27-07468-s001.zip › ijms-4430000-supplementary.pdf]

Supporting Information

Quantum Chemical Tailoring of Donor–Acceptor Organic Nanomedicines for Nonlinear Optical Performance and Theragnostic Applications: Molecular Descriptors, In Silico, and Ab Initio Investigations

Sehar Nadeem [1], Muhammad Usman Khan [1,*], Łukasz Szeleszczuk [2,*], Dariusz Maciej Pisklak [2], Marcin Gackowski [3], Salah Knani [4], and Nadia Ayari [5]

[1] Department of Chemistry, University of Okara, Okara 56300, Pakistan; sehar.nadeem230@gmail.com
[2] Department of Organic and Physical Chemistry, Medical University of Warsaw, 1 Banacha Str., 02-097 Warsaw, Poland; dpisklak@wum.edu.pl
[3] Department of Toxicology and Bromatology, L. Rydygier Collegium Medicum in Bydgoszcz, Nicolaus Copernicus University in Torun, 2 Jurasza Str., 85-089 Bydgoszcz, Poland; marcin.gackowski@cm.umk.pl
[4] Center for Scientific Research and Entrepreneurship, Northern Border University, Arar 73213, Saudi Arabia; saleh.kenani@nbu.edu.sa
[5] Department of Physics, College of Science, Northern Border University, Arar 91431, Saudi Arabia; nadia.alayari@nbu.edu.sa
* Correspondence: usman.chemistry@gmail.com or usmankhan@uo.edu.pk (M.U.K.); lukasz.szeleszczuk@wum.edu.pl (Ł.S.)

Table S1: The frontier molecular orbital parameters in eV

Molecules	HOMO (eV)	LUMO (eV)	Energy gap (eV)
**FTC-3f**	-5.209	-3.252	1.957
**D**	-5.304	-3.459	1.845
**D1**	-5.399	-3.577	1.822
**D2**	-5.447	-3.736	1.712
**D3**	-5.517	-3.802	1.715
**D4**	-5.568	-3.867	1.701

Table S2: The percentage composition of different fragments of all designed compounds in HOMO and LUMO

Molecules	HOMO			LUMO		
	Acceptor	Spacer	Donor	Acceptor	Spacer	Donor
**FTC-3F**	17.6	15.4	67	64.4	21.6	14
**D**	13.4	14.3	72.2	65.5	21.4	13.1
**D1**	20.5	16.5	63	61	22.5	16.5
**D2**	20.6	16.4	63	98.1	1.1	0.8
**D3**	15.7	15.1	69.2	63.4	21.8	14.8
**D4**	16	14.9	69.1	71.5	13.6	14.9

Table S3 (a): The optical absorption parameters at B3LYP

Molecules	vertical excitation energies $E_{(ext)}$ (eV)	Experimental Wavelength λ_{max}	Theoretical Wavelengths λ_{max} (nm)	oscillator strengths (*f*)	Major electronic transitions
**FTC-3F**	1.898	728 nm	653.339	1.4617	HOMO→LUMO (100%)
**D**	1.802		688.075	1.2979	HOMO→LUMO (100%)
**D1**	1.840		673.900	1.5868	HOMO→LUMO (100%)
**D2**	1.830		677.620	1.6042	HOMO→L+1 (97%)
**D3**	1.739		713.045	1.4248	HOMO→LUMO (100%)
**D4**	1.733		715.431	1.4212	HOMO→LUMO (97%)
Checking NIR response in FTC-3F in THF solvent by using CPCM					
	**vertical excitation energies $E_{(ext)}$ (eV)**	**Experimental Wavelength λ_{max}**	**Theoretical Wavelengths λ_{max} (nm)**	**oscillator strengths (*f*)**	**Major electronic transitions**
**FTC-3F**	1.638	728 nm	756.97	1.6732	HOMO→LUMO (100%)

Table S3 (b): The transition parameters at hybrid functional CAM-B3LYP

Molecules	vertical excitation energies $E_{(ext)}$ (eV)	Wavelengths λ_{max} (nm)	oscillator strengths (*f*)	Major electronic transitions
**FTC-3F**	2.257	549.381	1.824	HOMO→LUMO (88%)
**D**	2.210	560.989	1.697	HOMO→LUMO (88%)
**D1**	2.122	584.252	1.873	HOMO→LUMO (90%)
**D2**	2.098	590.879	1.881	HOMO→LUMO (90%)
**D3**	2.053	604.035	1.747	HOMO→LUMO (90%)
**D4**	2.035	609.229	1.757	HOMO→LUMO (90%)

Table S4: The NBO parameters of all investigated compounds

Molecules	Donor (i)	Type	Acceptor (j)	Type	$E^{(2)}$	Ej.Ej (a.u)	Fi.j (a.u)
**FTC-3F**	C 5 - C 6	π	C 3 - C 4	π*	26.84	0.28	0.077
	C 20 - C 21	π	C 23 - C 24	π*	23.63	0.29	0.074
	O 26	LP (2)	C 27 - C 29	π*	36.24	0.34	0.104
**D**	C 5 - C 6	π	C 3 - C 4	π*	26.98	0.28	0.078
	N 7	LP (1)	C 5 - C 6	π*	49.83	0.26	0.105
	C 1 - C 2	π	C 5 - C 6	π*	19.83	0.27	0.069
**D1**	C 5 - C 6	π	C 3 - C 4	π*	27.17	0.27	0.077
	C 16 - C 17	π	C 18 - C 19	π*	20.94	0.27	0.069
	N 33	LP (1)	C 29 - C 32	σ*	12.58	1.01	0.101
**D2**	C 1 - C 2	π	C 5 - C 6	π*	19.72	0.27	0.069
	C 3 - C 4	π	C 16 - C 17	π*	22.30	0.27	0.072
	O 88	LP (3)	N 85 - O 89	π*	143.86	0.14	0.129
**D3**	C 3 - C 4	π	C 16 - C 17	π*	22.26	0.27	0.072
	C 5 - C 6	π	C 3 - C 4	π*	27.30	0.27	0.078
	N 83	LP (1)	C 20 - S 21	σ*	20.10	0.44	0.084
**D4**	C 1 - C 2	π	C 5 - C 6	π*	19.72	0.27	0.069
	C 3 - C 4	π	C 16 - C 17	π*	22.36	0.2	0.073
	O 25	LP (2)	C 26 - C 28	π*	31.44	0.33	0.096

Table S5: The β_{zzz}, β_{zxx}, β_{HRS}, and DR ratio of all designed compounds and comparative result of B3LYP and CAM-B3LYP/6-31+G(d,p), and CAM-B3LYP/6-31++G(d,p).

Molecules	β_{zzz}	β_{zxx}	β_{HRS}	DR
**FTC-3F**	2.12×10^1	9.94×10^2	2.97×10^4	4.53×10^4
**D**	-6.02×10^5	-2.29×10^4	3.40×10^4	6.91×10^2
**D1**	-1.09×10^5	-9.86×10^3	3.83×10^4	1.22×10^2
**D2**	-1.20×10^5	-5.54×10^3	4.26×10^4	4.68×10^2
**D3**	-1.41×10^5	-3.27×10^3	4.68×10^4	1.86×10^3
**D4**	-1.47×10^5	-2.43E+02	5.11×10^4	3.65×10^5

Comparative results of diffuse and hybrid functional

Molecule	B3LYP/6-31G(d,p)	CAM-B3LYP/6-31+G(d,p)	CAM-B3LYP/6-31++G(d,p)
**FTC-3F**	1.21×10^5	1.12×10^5	1.12×10^5
**D**	1.41×10^5	1.24×10^5	1.24×10^5
**D4**	1.50×10^5	1.66×10^5	1.65×10^5

Table S6: The NLO parameters at diffuse basis set

	B3LYP/6-31+G(d,p)			B3LYP/6-31++G(d,p)		
**Molecules**	**μ**	**α**	**β**	**μ**	**α**	**β**
**FTC-3f**	2.24×10^1	9.27×10^2	1.20×10^5	2.24×10^1	9.29×10^2	1.20×10^5
**D**	2.16×10^1	9.37×10^2	1.44×10^5	2.16×10^1	9.39×10^2	1.44×10^5
**D1**	2.41×10^1	1.02×10^3	1.25×10^5	2.41×10^1	1.02×10^3	1.24×10^5
**D2**	2.39×10^1	1.04×10^3	1.30×10^5	2.39×10^1	1.04×10^3	1.30×10^5
**D3**	2.37×10^1	1.04×10^3	1.48×10^5	2.37×10^1	1.04×10^3	1.48×10^5
**D4**	2.68×10^1	1.06×10^3	1.53×10^5	2.68×10^1	1.06×10^3	1.53×10^5

Table S7: The binding affinity and dock score with proteins

4F5S			2W3L		
**Molecules**	**S**	**RMSD**	**Molecules**	**S**	**RMSD**
**FTC-3F**	-10.4779	2.885067	**FTC-3F**	-8.4095	1.996299
**D**	-7.05115	2.24755	**D**	-8.50128	1.158641
**D1**	-9.45993	2.817412	**D1**	-7.81458	1.757575
**D2**	-8.99924	3.940552	**D2**	-8.54776	1.448046
**D3**	-9.52319	3.996611	**D3**	-7.05644	2.168166
**D4**	-9.64642	3.270458	**D4**	-8.01366	2.555974

Table S8: The amino acid and distance and their interaction in case of both proteins **4F5S** and **2W3L**

**FTC-3F**			**FTC-3F**		
**Amino acids**	**Distance**	**Type**	**Amino acids**	**Distance**	**Types**
ASN404	1.94582	Conventional Hydrogen Bond;Halogen (Fluorine)	ARG8 8	3.03973	Conventional Hydrogen Bond
ASN404	2.47762	Conventional Hydrogen Bond;Halogen (Fluorine)	ARG8 8	2.7581	Conventional Hydrogen Bond;Halogen (Fluorine)
GLY401	2.46441	Carbon Hydrogen Bond;Halogen (Fluorine)	ARG8 8	2.78989	Conventional Hydrogen Bond;Halogen (Fluorine)
GLY401	2.79561	Carbon Hydrogen Bond;Halogen (Fluorine)	GLY7 7	2.61884	Carbon Hydrogen Bond

LEU531	2.898	Pi-Sigma	GLU95	3.58742	Pi-Anion
TYR400	5.1925	Pi-Sulfur	PHE63	5.24673	Pi-Pi T-shaped
LEU574	4.30049	Alkyl	MET74	4.30625	Alkyl
VAL575	4.45328	Alkyl	VAL92	4.0917	Alkyl
LEU531	4.98709	Alkyl	PHE63	5.29307	Pi-Alkyl
TYR400	4.36108	Pi-Alkyl	ALA108	4.8787	Pi-Alkyl
PHE501	4.8686	Pi-Alkyl	**D**		
PHE506	4.0106	Pi-Alkyl	ARG105	2.41697	Conventional Hydrogen Bond
PHE508	5.01166	Pi-Alkyl	ARG105	2.96335	Conventional Hydrogen Bond;Halogen (Fluorine)
HIS534	5.20248	Pi-Alkyl	ARG105	2.61965	Conventional Hydrogen Bond;Halogen (Fluorine)
ALA527	5.28612	Pi-Alkyl	LEU96	2.36936	Carbon Hydrogen Bond;Halogen (Fluorine)
VAL546	4.97799	Pi-Alkyl	GLY77	2.48584	Carbon Hydrogen Bond
LYS524	5.08872	Pi-Alkyl	GLY77	3.05599	Carbon Hydrogen Bond
LEU528	5.13617	Pi-Alkyl	GLU95	2.96731	Halogen (Fluorine)
MET547	3.82637	Pi-Alkyl	GLU95	2.84608	Halogen (Fluorine)
VAL551	4.86597	Pi-Alkyl	LEU96	3.59383	Halogen (Fluorine)
VAL551	4.17274	Pi-Alkyl	LEU96	2.88738	Halogen (Fluorine)
D			ASP99	3.39842	Halogen (Fluorine)
ASN404	1.94582	Conventional Hydrogen Bond;Halogen (Fluorine)	ASP99	3.42381	Halogen (Fluorine)
ASN404	2.47762	Conventional Hydrogen	ARG105	3.17141	Halogen (Fluorine)

		Bond;Halogen (Fluorine)			
GLY401	2.46441	Carbon Hydrogen Bond;Halogen (Fluorine)	VAL92	5.20149	Pi-Alkyl
GLY401	2.79561	Carbon Hydrogen Bond;Halogen (Fluorine)	LEU96	5.26736	Pi-Alkyl
LEU531	2.898	Pi-Sigma	ARG105	4.97212	Pi-Alkyl
TYR400	5.1925	Pi-Sulfur	ALA108	5.3367	Pi-Alkyl
LEU574	4.30049	Alkyl	**D1**		
VAL575	4.45328	Alkyl	ARG105	2.3889	Conventional Hydrogen Bond;Halogen (Fluorine)
LEU531	4.98709	Alkyl	ARG105	2.0647	Conventional Hydrogen Bond;Halogen (Fluorine)
TYR400	4.36108	Pi-Alkyl	ARG98	2.81276	Carbon Hydrogen Bond
PHE501	4.8686	Pi-Alkyl	LEU96	3.30692	Halogen (Fluorine)
PHE506	4.0106	Pi-Alkyl	ARG105	3.40227	Halogen (Fluorine)
PHE508	5.01166	Pi-Alkyl	ALA108	3.59501	Halogen (Fluorine)
HIS534	5.20248	Pi-Alkyl	ARG66	3.16135	Pi-Donor Hydrogen Bond
ALA527	5.28612	Pi-Alkyl	ARG66	2.59151	Pi-Donor Hydrogen Bond
VAL546	4.97799	Pi-Alkyl	TYR67	5.78865	Pi-Pi T-shaped
LYS524	5.08872	Pi-Alkyl	ALA108	4.39875	Alkyl
LEU528	5.13617	Pi-Alkyl	ARG69	4.65202	Alkyl
MET547	3.82637	Pi-Alkyl	PHE71	4.12823	Pi-Alkyl
VAL551	4.86597	Pi-Alkyl	LEU96	5.32916	Pi-Alkyl
VAL551	4.17274	Pi-Alkyl	**D2**		

D1			ASP99	2.19679	Conventional Hydrogen Bond
TYR400	2.81206	Conventional Hydrogen Bond	ARG105	2.79484	Conventional Hydrogen Bond
ASN404	2.73484	Conventional Hydrogen Bond;Halogen (Fluorine)	ARG105	2.45545	Conventional Hydrogen Bond;Halogen (Fluorine)
ASN404	2.71396	Conventional Hydrogen Bond	ARG105	2.78611	Conventional Hydrogen Bond
GLU548	3.01766	Carbon Hydrogen Bond	ARG105	2.35435	Conventional Hydrogen Bond;Halogen (Fluorine)
THR578:OG1	2.51458	Carbon Hydrogen Bond	ARG105	2.3082	Conventional Hydrogen Bond;Halogen (Fluorine)
TYR400	5.2265	Pi-Sulfur	ARG105	3.47038	Halogen (Fluorine)
PHE550	4.27802	Pi-Pi Stacked	ARG66	2.5663	Pi-Donor Hydrogen Bond
TYR400	4.73537	Pi-Pi T-shaped	ARG66	3.20424	Pi-Donor Hydrogen Bond
VAL546	5.59711	Amide-Pi Stacked	PHE63	5.23935	Pi-Pi T-shaped
LEU531	4.40069	Alkyl	ALA108	5.41317	Pi-Alkyl
LEU574	4.33113	Alkyl	**D3**		
VAL575	4.83154	Alkyl	ASN102	2.05863	Conventional Hydrogen Bond;Halogen (Fluorine)
TYR400	4.75735	Pi-Alkyl	ARG105	2.41001	Conventional Hydrogen Bond
PHE506	3.82269	Pi-Alkyl	GLY104	2.56052	Carbon Hydrogen Bond;Halogen (Fluorine)
PHE508	4.73967	Pi-Alkyl	GLU95	2.95814	Halogen (Fluorine)
PHE553	5.04997	Pi-Alkyl	ASP99	3.60164	Halogen (Fluorine)
ALA527	4.62817	Pi-Alkyl	ASP99	3.66427	Halogen (Fluorine)

LEU531	5.24481	Pi-Alkyl	ASN1<br>02	3.30036	Halogen (Fluorine)
LYS524	4.64115	Pi-Alkyl	ASN1<br>02	2.99654	Halogen (Fluorine)
MET547	4.34297	Pi-Alkyl	GLY1<br>04	3.64106	Halogen (Fluorine)
VAL551	5.28518	Pi-Alkyl	ASP99	3.41594	Pi-Anion
D2			MET7<br>4	2.7482	Pi-Sigma
GLY401	2.5191	Conventional Hydrogen Bond	MET7<br>4	4.76509	Alkyl
GLY401	2.65517	Carbon Hydrogen Bond	LEU78	5.35375	Alkyl
GLY401	2.34296	Carbon Hydrogen Bond	ARG1<br>05	4.43364	Alkyl
MET547	3.13815	Halogen (Fluorine)	**D4**		
ASP555	3.23033	Halogen (Fluorine)	ARG8<br>8	2.60359	Conventional Hydrogen Bond;Halogen (Fluorine)
ASP555	3.29079	Halogen (Fluorine)	ARG8<br>8	2.93302	Conventional Hydrogen Bond;Halogen (Fluorine)
ASP555	3.08109	Halogen (Fluorine)	GLY7<br>7	2.82969	Carbon Hydrogen Bond
LEU531	2.47434	Pi-Sigma	GLY7<br>7	3.05862	Carbon Hydrogen Bond
LEU531	4.59953	Alkyl	LEU78	2.66698	Carbon Hydrogen Bond;Halogen (Fluorine)
LEU574	4.31133	Alkyl	ASP99<br>:OD2	2.51595	Carbon Hydrogen Bond
VAL551	4.46781	Alkyl	ASP99<br>:OD1	2.97643	Carbon Hydrogen Bond
PHE501	5.47095	Pi-Alkyl	MET7<br>4	2.80859	Halogen (Fluorine)
PHE508	4.55485	Pi-Alkyl	GLY7<br>7	3.36597	Halogen (Fluorine)
HIS534	5.31864	Pi-Alkyl	ASP70	2.73014	Pi-Sigma
PHE550	5.04354	Pi-Alkyl	GLU7<br>3	4.32472	Amide-Pi Stacked

ALA527	5.10046	Pi-Alkyl	MET74	5.04227	Alkyl
VAL546	5.00279	Pi-Alkyl	LEU78	5.34055	Alkyl
LYS524	5.19311	Pi-Alkyl	VAL92	4.74514	Pi-Alkyl
LEU528	5.4021	Pi-Alkyl	MET74	4.79669	Pi-Alkyl
MET547	3.75441	Pi-Alkyl	MET74	5.46718	Pi-Alkyl
VAL551	3.72535	Pi-Alkyl			
VAL551	4.99673	Pi-Alkyl			
D3					
TYR400	2.81418	Conventional Hydrogen Bond			
TYR400	2.56554	Conventional Hydrogen Bond			
LYS524	2.34318	Conventional Hydrogen Bond			
LYS524	2.71582	Conventional Hydrogen Bond			
GLY398	2.67171	Carbon Hydrogen Bond			
VAL551	2.89628	Carbon Hydrogen Bond			
LYS524:O	2.80303	Carbon Hydrogen Bond			
LYS524:O	2.42232	Carbon Hydrogen Bond			
TYR400	4.78843	Pi-Sulfur			
ALA527	3.4747	Alkyl			
LEU528	4.1294	Alkyl			
LEU531	4.52143	Alkyl			
VAL546	4.52255	Alkyl			
MET547	4.10549	Alkyl			
PHE508	5.2508	Pi-Alkyl			
PHE550	4.59818	Pi-Alkyl			
LYS524	4.88667	Pi-Alkyl			
LEU528	5.29146	Pi-Alkyl			
MET547	4.12487	Pi-Alkyl			

VAL551	4.83892	Pi-Alkyl			
D4					
TYR400	2.72479	Conventional Hydrogen Bond			
ASN404	2.66538	Conventional Hydrogen Bond			
GLY401	2.74875	Carbon Hydrogen Bond			
THR578: OG1	2.82368	Carbon Hydrogen Bond			
ASP555	3.58891	Halogen (Fluorine)			
ASP555	3.59268	Halogen (Fluorine)			
PHE550	4.21863	Pi-Pi Stacked			
ALA527	4.30833	Amide-Pi Stacked			
LEU531	4.36702	Alkyl			
LEU574	3.76865	Alkyl			
VAL575	4.42291	Alkyl			
VAL551	4.31941	Alkyl			
PHE506	3.03299	Pi-Alkyl			
ALA527	4.15849	Pi-Alkyl			
LEU531	5.37201	Pi-Alkyl			
LYS524	5.20892	Pi-Alkyl			
MET547	4.17881	Pi-Alkyl			
VAL551	3.95459	Pi-Alkyl			

Table S9: The molecular descriptors calculated by using frontier molecular orbital parameters

**Molecules**	**IP (Ionization potential)**	**EA (Electron affinity)**	**X (Electro-negativity)**	**η (Chemical Hardness)**	**σ (Softness)**	**μ (Chemical Potential)**	**ω (Electrophilicity)**
**FTC-3f**	5.209	3.252	4.230	0.978	0.511	-4.230	9.147
**D**	5.304	3.459	4.381	0.922	0.542	-4.381	10.406
**D1**	5.399	3.577	4.488	0.911	0.549	-4.488	11.055
**D2**	5.447	3.736	4.592	0.856	0.584	-4.592	12.319
**D3**	5.517	3.802	4.659	0.857	0.583	-4.659	12.661
**D4**	5.568	3.867	4.718	0.851	0.588	-4.718	13.083

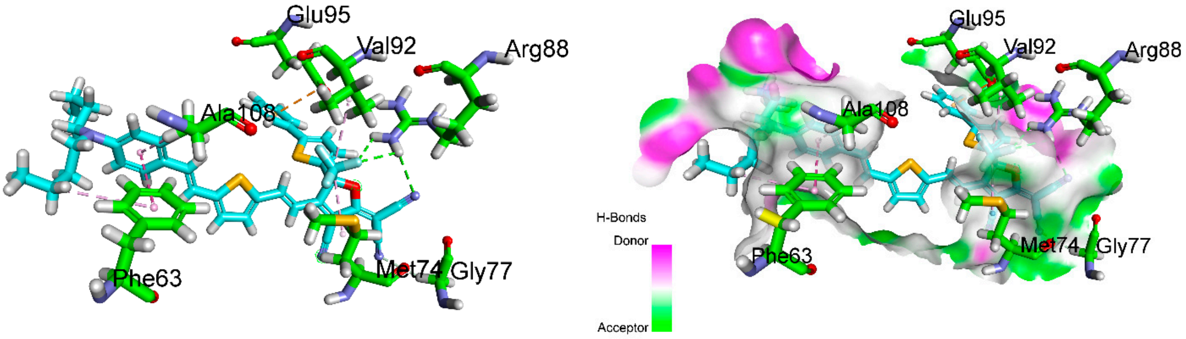
Glu95
Val92
Arg88
Ala108
Phe63
Met74
Gly77
H-Bonds
Donor
Acceptor

FTC-3F

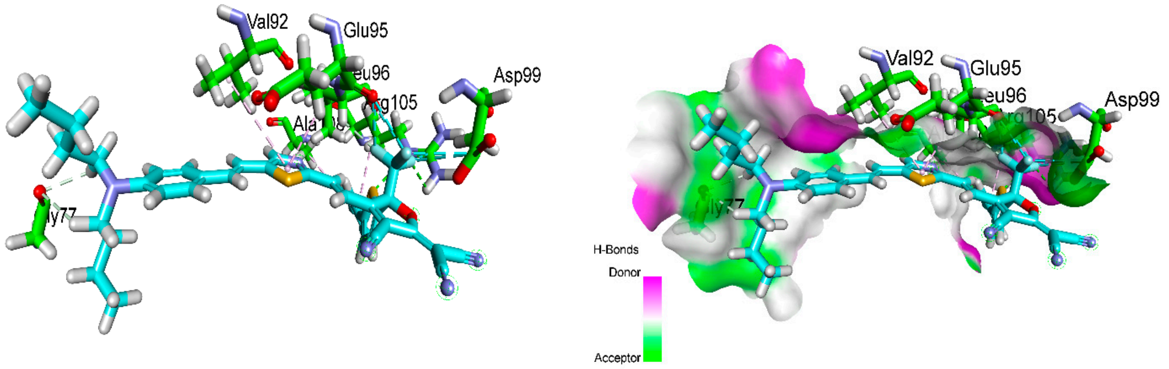
Val92
Glu95
Asp99
H-Bonds
Donor
Acceptor

D

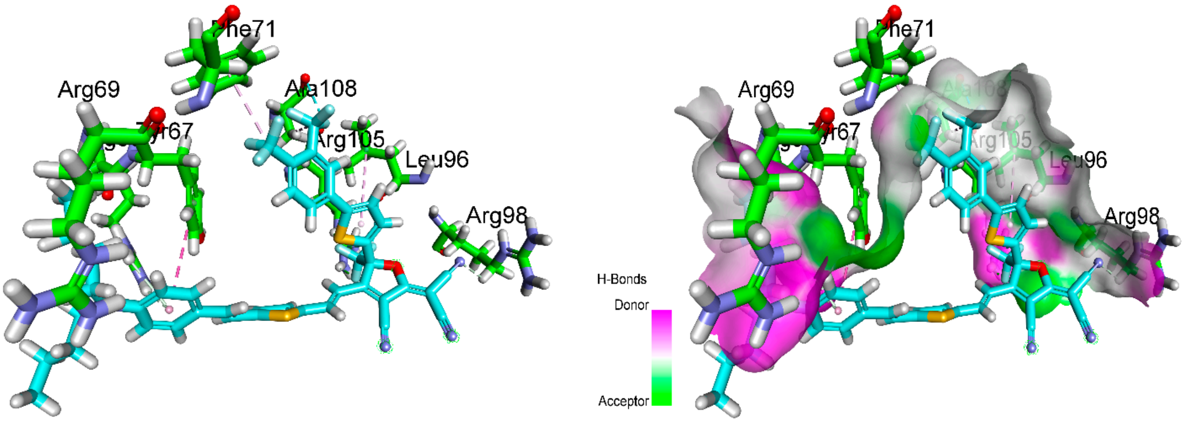
Arg69
Ala108
Leu96
Arg98
Arg105
H-Bonds
Donor
Acceptor

D1

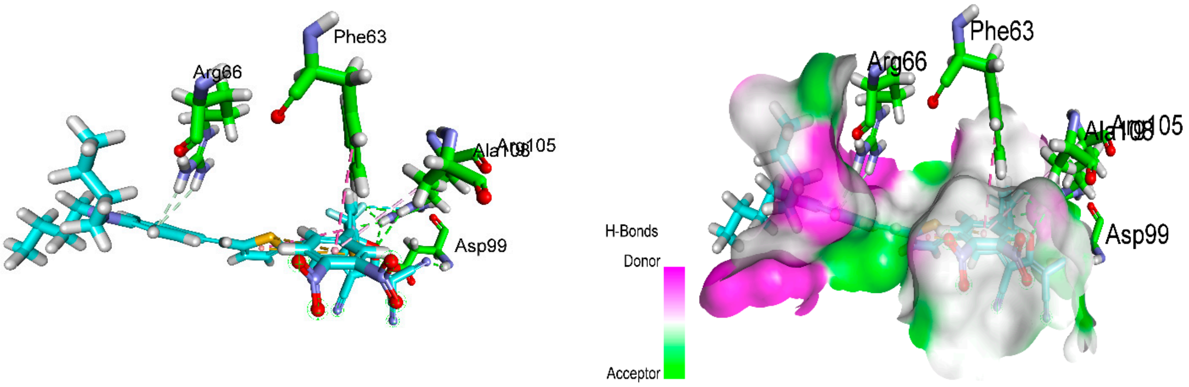

D2

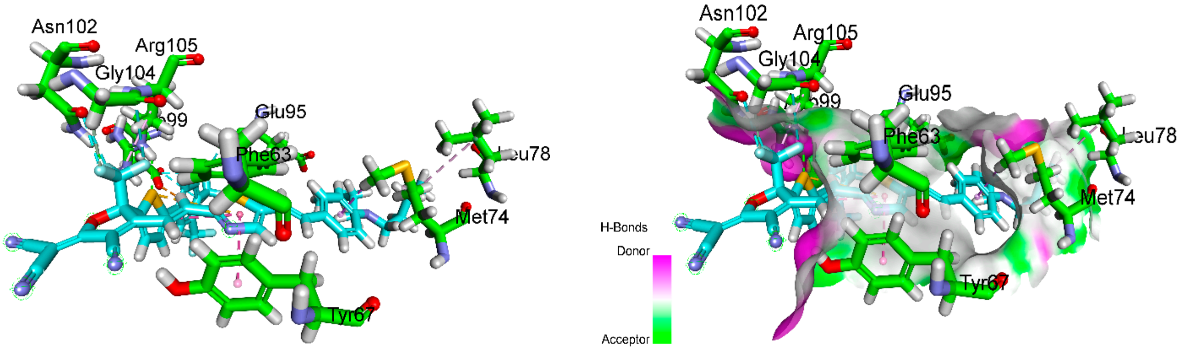

D3

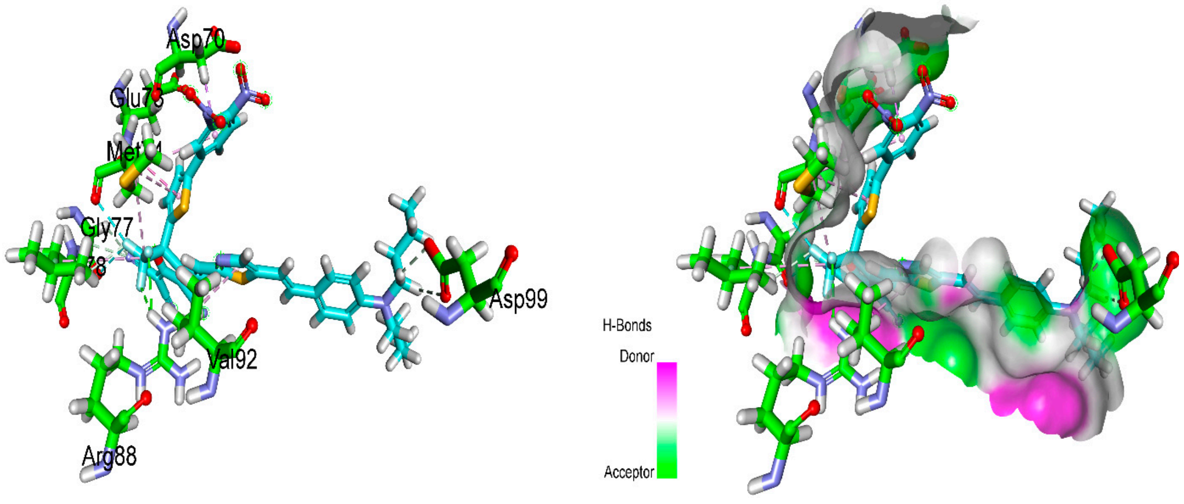

D4

Figure S1: The 3D and H-bond surface in case of protein (2W3L)

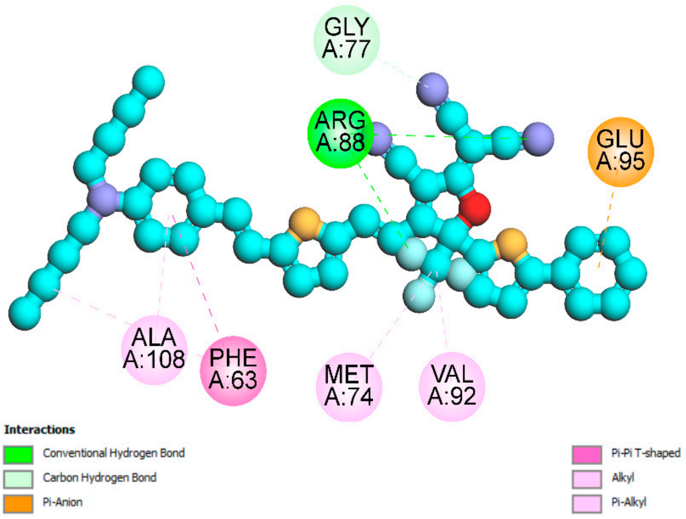
GLY A:77
ARG A:88
GLU A:95
ALA A:108
PHE A:63
MET A:74
VAL A:92
Interactions
Conventional Hydrogen Bond
Carbon Hydrogen Bond
Pi-Anion
Pi-Pi T-shaped
Alkyl
Pi-Alkyl

FTC-3F

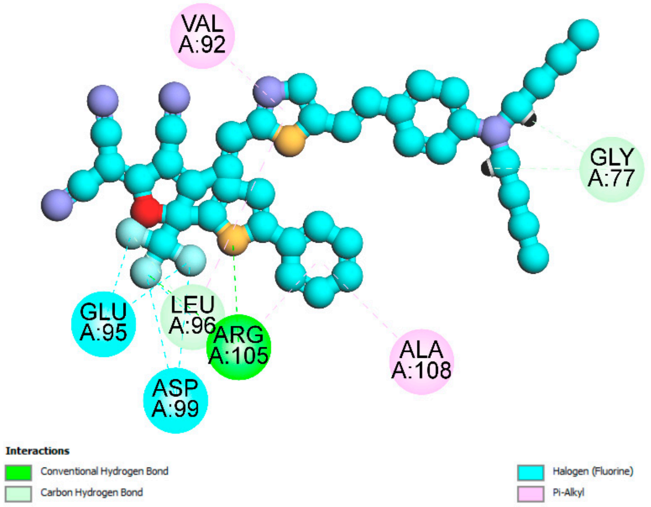
VAL A:92
GLY A:77
GLU A:95
LEU A:96
ARG A:105
ASP A:99
ALA A:108
Interactions
Conventional Hydrogen Bond
Carbon Hydrogen Bond
Halogen (Fluorine)
Pi-Alkyl

D

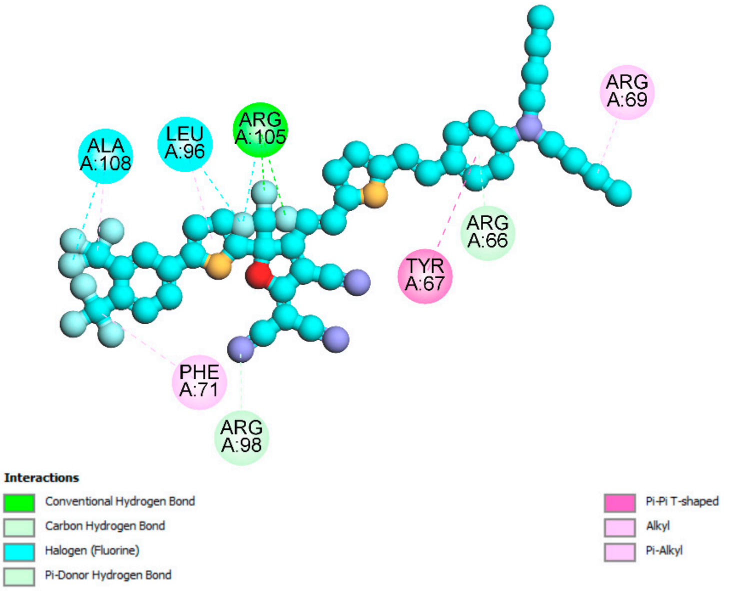
ALA A:108
LEU A:96
ARG A:105
ARG A:69
ARG A:66
TYR A:67
PHE A:71
ARG A:98
Interactions
Conventional Hydrogen Bond
Carbon Hydrogen Bond
Halogen (Fluorine)
Pi-Donor Hydrogen Bond
Pi-Pi T-shaped
Alkyl
Pi-Alkyl

D1

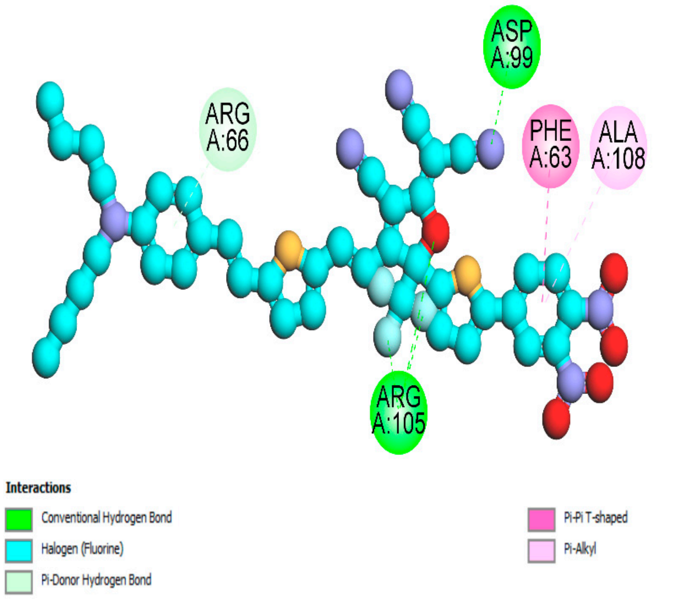
ASP A:99
ARG A:66
PHE A:63
ALA A:108
ARG A:105
Interactions
Conventional Hydrogen Bond
Halogen (Fluorine)
Pi-Donor Hydrogen Bond
Pi-Pi T-shaped
Pi-Alkyl

D2

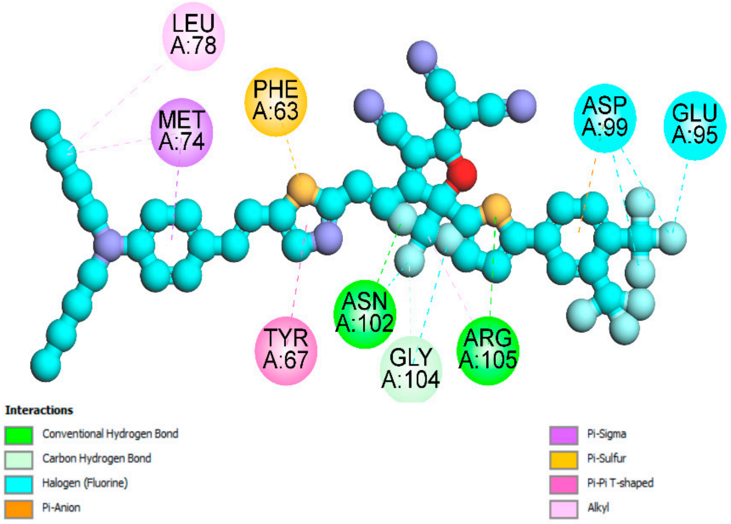

D3

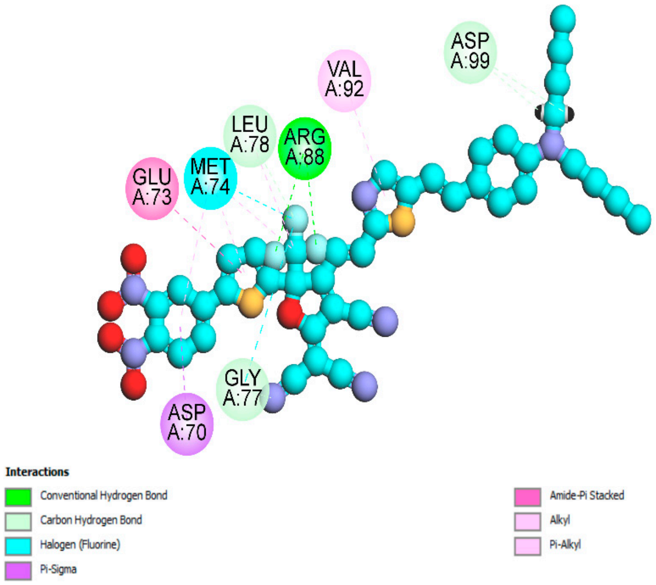

D4

Figure S2: The 2D interaction plots of protein and legend
